# Specialized Metabolites from Ribosome Engineered Strains of *Streptomyces clavuligerus*

**DOI:** 10.3390/metabo11040239

**Published:** 2021-04-13

**Authors:** Arshad Ali Shaikh, Louis-Felix Nothias, Santosh K. Srivastava, Pieter C. Dorrestein, Kapil Tahlan

**Affiliations:** 1 Department of Biology, Memorial University of Newfoundland, St. John’s, NL A1B 3X9, Canada; aashaikh@mun.ca (A.A.S.); sksrivastava@mun.ca (S.K.S.); 2 Collaborative Mass Spectrometry Innovation Center, Skaggs School of Pharmacy and Pharmaceutical Sciences, University of California, San Diego, La Jolla, CA 92093, USA; lnothiasscaglia@health.ucsd.edu (L.-F.N.); pdorrestein@health.ucsd.edu (P.C.D.)

**Keywords:** *Streptomyces*, specialized metabolites, ribosome engineering, ribosome recycling factor, metabolomics, global molecular networking

## Abstract

Bacterial specialized metabolites are of immense importance because of their medicinal, industrial, and agricultural applications. *Streptomyces clavuligerus* is a known producer of such compounds; however, much of its metabolic potential remains unknown, as many associated biosynthetic gene clusters are silent or expressed at low levels. The overexpression of ribosome recycling factor (*frr*) and ribosome engineering (induced *rpsL* mutations) in other *Streptomyces* spp. has been reported to increase the production of known specialized metabolites. Therefore, we used an overexpression strategy in combination with untargeted metabolomics, molecular networking, and in silico analysis to annotate 28 metabolites in the current study, which have not been reported previously in *S. clavuligerus*. Many of the newly described metabolites are commonly found in plants, further alluding to the ability of *S. clavuligerus* to produce such compounds under specific conditions. In addition, the manipulation of *frr* and *rpsL* led to different metabolite production profiles in most cases. Known and putative gene clusters associated with the production of the observed compounds are also discussed. This work suggests that the combination of traditional strain engineering and recently developed metabolomics technologies together can provide rapid and cost-effective strategies to further speed up the discovery of novel natural products.

## 1. Introduction

*Streptomyces* are Gram-positive filamentous bacteria that undergo sporulation and comprise the largest genus of the phylum *Actinobacteria* [1]. They are found in various natural environments, such as soil and marine sediments. In addition, *Streptomyces* genomes range from 5 to 12.5 Mbp in size and contain an average of 39 biosynthetic gene clusters (BGCs) [2], responsible for the production of numerous and diverse specialized (or secondary) metabolites (SMs), many of which are used in medicine, industry, and agriculture [1,2]. *Streptomyces clavuligerus* is the industrial producer of clavulanic acid, a β-lactamase inhibitor used in combination with β-lactam antibiotics to treat certain otherwise-resistant bacterial infections [3]. In addition, *S. clavuligerus* is also known to produce cephamycin C [4], 5*S* clavams [5], naringenin [6,7], holomycin [8], and tunicamycins [8]. Different genome sequencing studies have reported the presence of 49–58 SM BGCs in *S. clavuligerus* [9,10], most of which are thought to be cryptic (or silent) as the identities of their SM products remain unknown [10,11]. Activation of production or improved yields of these cryptic SMs can lead to the identification of new biosynthetic capabilities. For example, *S. clavuligerus* was recently shown to produce certain plant-associated metabolites [6,7], previously not thought to be synthesized by bacteria [6]. In addition, genes for the biosynthesis of some of these metabolites do not reside in defined BGCs, providing additional challenges for their identification and heterologous expression. Therefore, the use of more generalized approaches, such as the manipulation of global regulator genes and those involved in other core physiological processes, provides potential avenues for enhancing endogenous specialized metabolism in such situations [12].

Ribosome engineering has been used to manipulate SM production by targeting *rpsL* [13], which encodes the ribosomal protein S12 and is involved in maintaining translational accuracy [14]. Specific point mutations in *rpsL* are known to activate or enhance antibiotic production in *Streptomyces* by increasing protein synthesis and the stability of 70S ribosomes [13,15]. In addition, it was shown that some *rpsL* mutations in an environmental *Streptomyces* isolate activated the production of a novel antibiotic called piperidamycin, which is not produced by the host organism under normal laboratory conditions [16]. It has also been reported that certain *rpsL* mutations enhance protein synthesis during later stages of growth, which coincides with SM production in most microorganisms and could therefore be advantageous for biosynthesis [17,18]. Specific *rpsL* point mutations, including *rpsL*-K88E (also used in the current study), lead to the overproduction of oligomycin in *Streptomyces avermitilis* [19], actinorhodin in *Streptomyces coelicolor* [17] and *Streptomyces lividans* [20], undecylprodigiosin in *S. lividans* [21], actinomycin in *Streptomyces antibioticus* [19], and landomycin in *Streptomyces cyanogenus* S136 [22]. It has also been reported that the *rpsL*-K88E mutation in *S. coelicolor* leads to the increased expression of *frr* [17], which encodes the ribosome recycling factor responsible for the dissociation of ribosomal subunits after the termination of translation [23]. The engineered overexpression of *frr* increases the production of the nucleoside antibiotic toyocamycin [24] and the insecticide/antihelmintic avermectin [25] in *Streptomyces diastatochromogenes* and *S. avermitilis*, respectively, by enhancing the expression of specific regulatory and biosynthetic genes. In addition, *frr* overexpression also promotes cellular proliferation in *S. avermitilis* [25] and protein synthesis during late growth phase in *S*. *diastatochromogenes* [24]. Therefore, some studies have suggested that ribosome engineering and *frr* overexpression might influence specialized metabolism in *Streptomyces* using similar mechanisms.

Ribosome engineering has been applied to other bacteria and fungi [13], but most studies only examined its influence on the production of a single or a known metabolite. In addition, the link between certain *rpsL* mutations and *frr* overexpression suggests that the two processes might activate the production of similar metabolites, but the influence of such ribosomal manipulations on overall specialized metabolism in *Streptomyces* is not known. Therefore, we overexpressed *frr*, *rpsL*, and three different *rpsL* variants (K88E, L90K, and R94G) in *S. clavuligerus* separately for untargeted metabolomics using liquid chromatography and tandem mass spectrometry (LC-MS/MS). The effect of such manipulations on the *S. clavuligerus* specialized metabolome was analyzed using a combination of global natural products social molecular networking (GNPS) [26] and in silico metabolite annotation, which included network annotation propagation (NAP) [27]. The results obtained further allude to the metabolic capabilities of this industrially important organism.

## 2. Results and Discussion

### 2.1. Overview of Metabolomics Analysis

**Figure 1:**
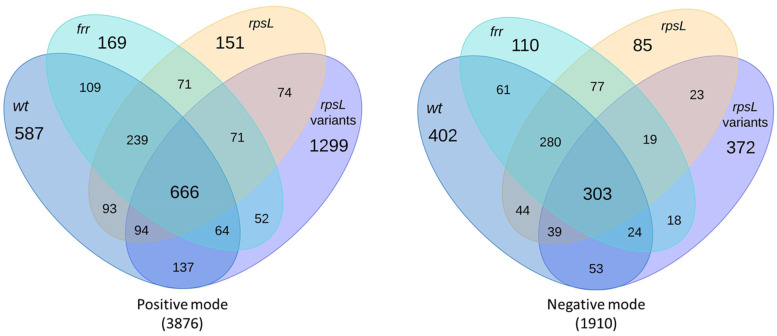
Overview of the numbers of spectral features detected in wild-type *S. clavuligerus* (wt) and strains overexpressing *frr*, *rpsL*, or *rpsL* variants (K88E, L90K, and R94G), respectively. The total number of spectral features detected in each ionization mode is indicated in parentheses.

*S. clavuligerus* gene overexpression strains were prepared by introducing integrative plasmids containing *frr*, *rpsL*, or *rpsL* variants under the control of a strong constitutive promoter (Table 1). Wild-type *S. clavuligerus* and the engineered strains were cultured on five different media and subjected to untargeted metabolomics using both positive and negative ionization modes in LC-MS/MS. A total of 5786 spectral features were detected during the analysis, some of which were only present in extracts from *S. clavuligerus* strains overexpressing either *frr*, *rpsL*, or *rpsL* variants (Figure 1). In addition, the number of spectral features detected in the positive ionization mode in extracts from *S. clavuligerus* strains overexpressing any individual *rpsL* variant was greater than those overexpressing *frr* or *rpsL* (Figure 1), suggesting metabolic modulation due to ribosomal protein S12 modification. Differentially produced metabolites were subjected to more detailed in silico structural analysis using recently developed methodologies [27], leading to the identification of 28 putative metabolites not reported in *S. clavuligerus* previously: 2 from library matches in GNPS and 26 using predicted fragmentation patterns in NAP (Table 2). Overall, the experimental spectra obtained in the current study mostly matched the generated or predicted spectra (Figures S1–S7), except for the putative organonitrogen compounds where certain high-intensity peaks differed (Figure S8). Therefore, it is possible that in some cases, the actual metabolites might vary or could be isomers of those presented. These 28 putative metabolites along with their known or predicted BGCs are discussed below to highlight important findings.

### 2.2. Triterpenoids and Derivatives

**Figure 2:**
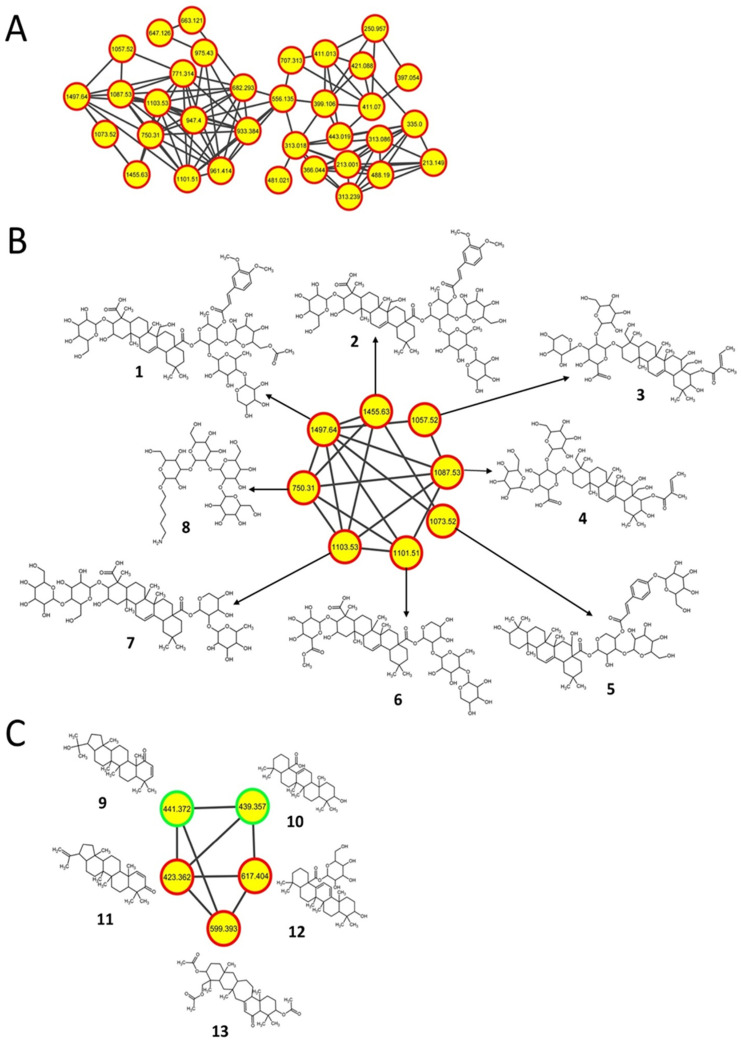

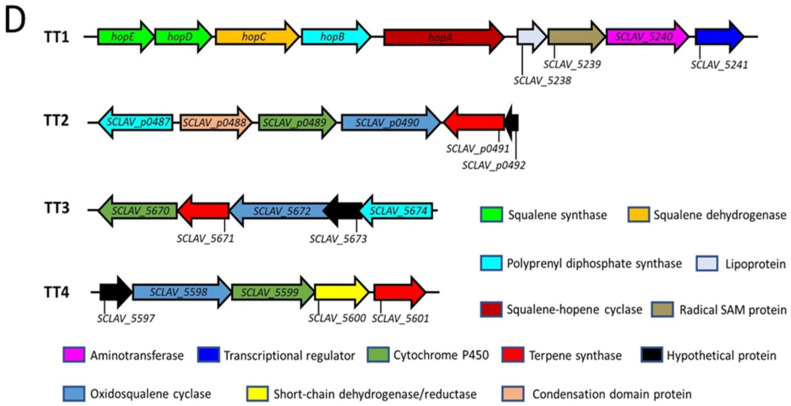
Triterpenoid molecular networks and related biosynthetic gene clusters (BGCs) corresponding to metabolites detected only in *S. clavuligerus* strains overexpressing different *rpsL* variants (K88E, L90K, and R94G). (**A**) Negative-mode molecular network comprising unknown and predicted triterpenoids from the current study. (**B**) Predicted structures of saponin triterpenoids annotated with network annotation propagation (NAP). (**C**) Structural prediction of another triterpenoid molecular network detected using positive-mode ionization. (**A**–**C**) Each node depicts a mass spectrum (labeled with *m/z* of the respective precursor mass) and edges represent the relationship between different nodes. (**B**,**C**) Top-ranked NAP-consensus structural predictions (red boundary) and those annotated by spectral library matching (green boundary) present in *S. clavuligerus* cultures overexpressing different *rpsL* variants (K88E, L90K, and R94G) (yellow fill) are shown. (**D**) BGCs proposed to be associated with the production of such metabolites in *S. clavuligerus*. TT1 is responsible for the production of hopanoids, whereas the products of the other three BGCs are not known.

Triterpenoids are widely distributed in both edible and medicinal plants [32], and some of them possess antibacterial [33], antiviral [34], anti-inflammatory [35], and antitumor [36] properties. The production of such metabolites, which include hopanoids (a triterpenoid subclass), has also been reported in some *Streptomyces* spp. previously [37,38,39]. In the current study, a molecular network containing predicted triterpenoid glycosides with high *m/z* values was detected only in *S. clavuligerus* strains overexpressing *rpsL* variants (Figure 2A,B). The metabolites were annotated as pentacyclic triterpenoids, which include oleanolic acid (**10**, *m/z* 439.357) and related glycosylated derivatives (also called saponins) such as securioside A (**1**, *m/z* 1497.64), CID: 56924794 (**2**, *m/z* 1455.63), eryngioside E (**3**, *m/z* 1057.52), CID: 10582011 (**4**, *m/z* 1087.53), tragopogonsaponin Q (**5**, *m/z* 1073.52), SN00394245 (**6**, *m/z* 1101.51), CID: 101205416 (**7**, *m/z* 1103.53), CID: 10603865 (**12**, *m/z* 617.404), and SN00379882 (**13**, *m/z* 599.393) (Figure 2B,C; Figures S1 and S2; Table 2). These putative triterpenoids are comprised of either an oleanolic acid or a β-amyrin core except for **13**, which contains a 7-membered ring as part of its pentacyclic core (Figure 2B,C). A mannooligosaccharide derivative unrelated to the triterpenoids but containing a carbohydrate core similar to the glycosides was also detected as part of the network in the same *S. clavuligerus* strains (**8**, *m/z* 750.31), suggesting that such glycosylation might be somehow upregulated under the given conditions (Figure 2B and Figure S1; Table 2).

The hopanoids 22-Hydroxy-2-hopen-1-one (**9**, *m/z* 441.372) and glochidone (**11**, *m/z* 423.362) were also detected in *S. clavuligerus* strains overexpressing *rpsL* variants (Figure 2C and Figure S2; Table 2). Triterpene and hopane biosynthesis occurred using the mevalonic acid pathway, which has been elucidated in plants (Figure S9) [40]. In addition, the BGC responsible for hopanoid production in *Streptomyces* is known (TT1, Figure 2D) [38,39]. In the current study, additional triterpenoid-like BGCs (TT2-4, Figure 2D) in *S. clavuligerus* were identified based on genes homologous to those present in the known hopane BGC and genes encoding enzymes catalyzing essential reactions in the biosynthesis of triterpenes in plants (Figure S9). The respective BGCs contain all the genes required for triterpenoid production, with the exception of TT4 (*SCLAV_5597* to *SCLAV_5601*), which is missing a polyprenyl diphosphate synthase (Figure 2D and Figure S9). Therefore, it is possible that **9** and **11** are produced by the known hopane BGC (TT1) present in *S. clavuligerus*, whereas the other three proposed BGCs (TT2-4) are responsible for the biosynthesis of triterpenoid **10** and the glycosylated triterpenoids (Figure 2D). Currently, the BGC involved in glycosylated triterpenoid production is not known, but a gene encoding a putative glycosyltransferase (*SCLAV_5660*) is situated next to TT3, which might suggest that it is involved in the process. It also seems that the overexpression of the *rpsL* variants increased or induced the production of the respective metabolites associated with these BGCs (Figure 2D), as they were not detected in other *S. clavuligerus* strains from the current study.

### 2.3. Flavonoids and Derivatives

**Figure 3:**
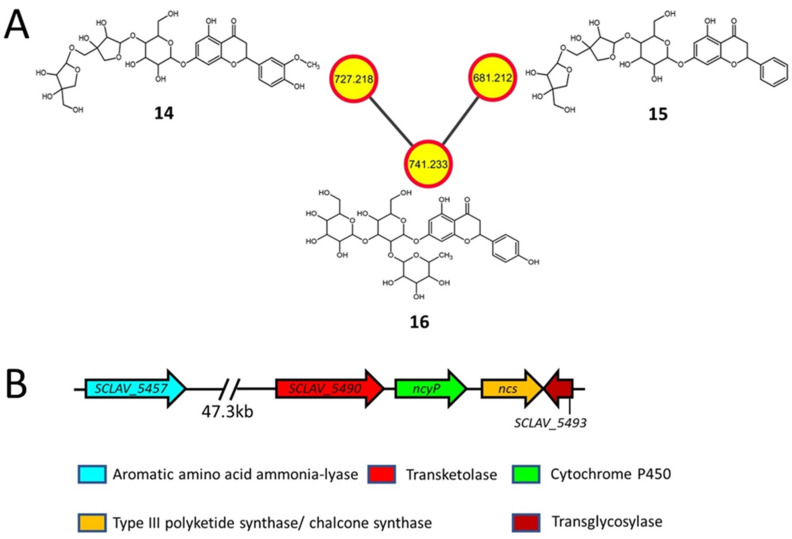
Molecular network (negative ionization mode) and associated biosynthetic gene cluster (BGC) for flavonoid glycosides detected in *S. clavuligerus* strains overexpressing the K88E variant of *rpsL* (**A**) In silico structure prediction of metabolites using GNPS-based molecular networking and network annotation propagation (NAP). Each node depicts a mass spectrum (labeled with *m/z* of the respective precursor mass) and edges represent the relationship between different nodes. The top-ranked NAP-consensus structural predictions (red boundary) present in the *S. clavuligerus* strain overexpressing the K88E variant of *rpsL* (yellow fill) are shown. (**B**) The proposed BGC in *S. clavuligerus* associated with the biosynthesis of flavonoid glycosides, including the previously reported genes required for naringenin (flavonoid) production.

Many plants also contain flavonoids and related glycosides with antibacterial [41], antifungal [42], anti-inflammatory [43], anti-plasmodial [44], and anticancer [45] properties, some of which were also detected during the current analysis. Viscumneoside V (**14**, *m/z* 727.218), CID: 42607862 (**15**, *m/z* 681.212) and monoglucosyl naringin (**16**, *m/z* 741.233) were detected only in the extracts from the *S. clavuligerus rpsL*-K88E strain (Figure 3A and Figure S3; Table 2). The perennial parasitic plant *Viscum angulatum*, used for the treatment of arthritis and hypertension [46], produces **14** and **15**, whereas the deglucosyl of **16** (or naringin) is produced by various citrus fruits and has many potential medicinal applications [47]. Previously it was thought that only plants produced naringenin, but recent studies have demonstrated its production in *S. clavuligerus* and other clavulanic acid producers, where the genes involved were also identified [6,7].

As no other flavonoid BGC or associated genes are present in *S. clavuligerus* [7,9], it is plausible that the naringenin BGC is also responsible for the production of the flavonoid glycosides detected in the current study (Figure 3B). The flavonoid cores of **14**, **15**, and **16** consist of homoeriodictyol, pinocembrin, and naringenin, respectively (Figure 3A and Figure S10). The known precursor for pinocembrin is phenylalanine [48], whereas naringenin is produced using tyrosine [6], and the enzymes encoded by *SCLAV_5457*, *ncs*, and *ncyP* (Figure 3B) could be involved in the formation of the flavonoid core using the described precursors, respectively (Figure S10). In plants, homoeriodictyol is synthesized using ferulic acid as a precursor [49], which is itself produced using tyrosine (Figure S10) [50]. Therefore, it is possible that a similar route could exist in *S. clavuligerus*, as the genes and an intermediate involved in ferulic acid formation were also detected in the current study (Figure S10). In addition, this species contains genes encoding a predicted transketolase (*SCLAV_5490*) required for the formation of the pentose sugars present in **14** and **15**, and a transglycosylase (*SCLAV_5493*) required for the modification of the flavonoid core. The detection of flavonoid glycosides in the *S. clavuligerus rpsL*-K88E overexpression strain suggests that an avenue to produce such metabolites in bacteria under laboratory conditions exists, which warrants further analysis.

### 2.4. Nucleoside Antibiotics

**Figure 4:**
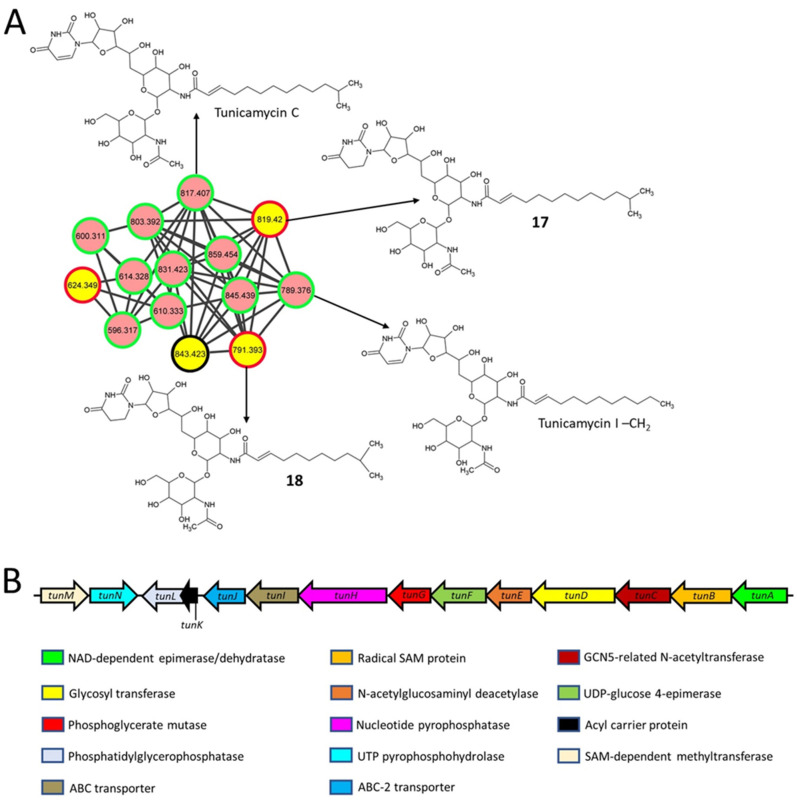
Molecular network (positive ionization mode) and associated biosynthetic gene cluster (BGC) for the tunicamycins and streptovirudins detected in *S. clavuligerus* strains. (**A**) Predicted structures of some metabolites using GNPS-based molecular networking and network annotation propagation (NAP). Each node depicts a mass spectrum (labeled with *m/z* of the respective precursor mass) and edges represent the relationship between different nodes. The top-ranked consensus structural predictions from NAP (red boundary) and GNPS (green boundary), or not predicted by both GNPS and NAP (black boundary) are shown. The pink nodes indicate the presence of the metabolites detected in both wild-type *S. clavuligerus* and the overexpression strains, whereas yellow nodes represent those detected only in strains overexpressing *frr*. Tunicamycins are included for comparison with the predicted structures. (**B**) The BGC is associated with the biosynthesis of tunicamycins and possibly that of the streptovirudins from *S. clavuligerus*.

Streptovirudins are nucleoside antibiotics and antivirals produced by *Streptomyces griseoflavus* subsp. *thuringiensis* [51], and some of them were also identified during the current analysis. Streptovirudin C1 (**17**, *m/z* 819.42) and streptovirudin A1 (**18**, *m/z* 791.393) are classified as series I streptovirudins [52] and were only detected in extracts from the *S. clavuligerus* strain overexpressing *frr* (Figure 4A and Figure S4; Table 2). Another metabolite (*m/z* 843.423) was also a part of the network (Figure 4A), but a structure could not be assigned using NAP. The streptovirudins (**17** and **18**) resemble the nucleoside antibiotic tunicamycin (Figure 4A), which is known to be produced by *S. clavuligerus* [8], and the associated BGC was identified in this organism (Figure 4B) [53]. Tunicamycins were also detected in the current study (Figure 4A) but are not discussed in detail as they have been reported previously [7,54]. In series I streptovirudins, the uracil (or uridine) moiety of tunicamycins is replaced by dihydrouracil (or dihydrouridine) [52]; otherwise, the metabolites are identical (Figure 4A). A closer examination of all reported *S. clavuligerus* genome sequences revealed that this organism only contains one nucleoside, SM BGC [7,9], i.e., the one involved in tunicamycin production. Therefore, our analysis suggests that the overexpression of *frr* (but not *rpsL* or its variants) in *S. clavuligerus* somehow leads to the production of streptovirudins using the biosynthetic machinery of tunicamycin. In *S. clavuligerus*, the tunicamycin BGC does not contain any gene encoding a reductase to explain the formation of streptovirudins from tunicamycin directly, as reported for other similar SMs [55,56], but the genes involved in converting free uracil into dihydrouracil are present elsewhere in the *S. clavuligerus* genome. Therefore, it is plausible that dihydrouracil is a precursor in the biosynthesis of the detected streptovirudins, a topic that needs further investigation.

### 2.5. Polycyclic Tetramate Macrolactams

**Figure 5:**
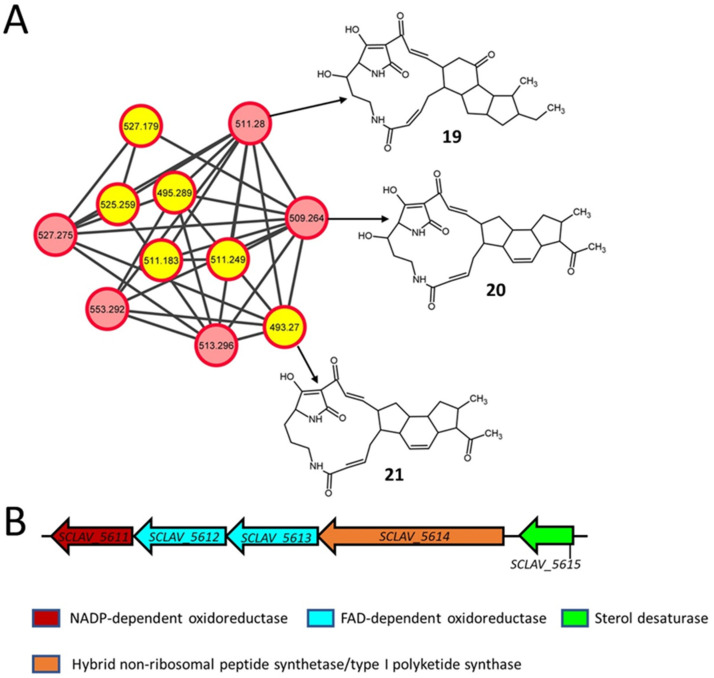
Molecular network (positive ionization mode) for polycyclic tetramate macrolactams (PTMs) present in different cultures from the current study and the associated biosynthetic gene cluster (BGC) in *S. clavuligerus*. (**A**) In silico structure prediction of the metabolites by GNPS-based molecular networking and network annotation propagation (NAP). Each node depicts a mass spectrum (labeled with *m/z* of the respective precursor mass) and edges represent the relationship between different nodes. The top-ranked NAP-consensus structural predictions (red boundary) for the metabolites present in all *S. clavuligerus* cultures, including wild-type (pink fill), and only in the different overexpression strains (yellow fill), are shown. (**B**) The proposed BGC is associated with the biosynthesis of PTMs detected in *S. clavuligerus*.

Polycyclic tetramate macrolactams (PTMs) are a class of SMs containing tetramic acid and a polycyclic carbocycle fused to macrolactams, which are widely produced by actinomycetes and other bacteria [57,58,59,60]. They are of therapeutic interest because of their antibacterial [61], antifungal [62], antiprotozoal [63], and antitumor [64] properties. Maltophilin (**19**, *m/z* 511.28) and clifednamide B (**20**, *m/z* 509.264) were detected in all the *S. clavuligerus* strains in the current study, whereas clifednamide A (**21**, *m/z* 493.27) was only detected in the strains overexpressing *rpsL* variants (Figure 5A and Figure S5; Table 2). Maltophilin was first reported in *Stenotrophomonas maltophilia* and is a broad-spectrum antifungal [62], whereas the clifednamides were produced by an environmental *Streptomyces* isolate (sp. JV178) [65]. The clifednamides are derivatives of ikarugamycin, another PTM that has antimicrobial [63], antiprotozoal [63], and anticancer properties [64]. Even though a BGC for PTMs was identified in *S. clavuligerus* previously (Figure 5B) [59], the production of these metabolites has not been reported in this organism to date. Therefore, our ability to detect putative PTMs in *S. clavuligerus* suggests that the production of such metabolites is possible under laboratory conditions.

The biosynthesis of maltophilin requires genes encoding a hybrid non-ribosomal peptide synthetase (NRPS)/type I polyketide synthase (PKS), NADP or FAD-dependent oxidoreductase and sterol desaturase [66], all of which are present in the single PTM BGC from *S. clavuligerus* (Figure 5B) and can account for the production of the metabolite. However, a cytochrome P450 enzyme is required for the biosynthesis of clifednamide A from ikarugamycin (Figure S11) [67], which is not encoded in the *S. clavuligerus* PTM BGC. It is possible that another cytochrome P450 located elsewhere in the chromosome of *S. clavuligerus* could perform this function, as noted in the biosynthesis of other SMs [68] and exemplified by a promiscuous sterol desaturase from *Lysobacter capsici* DSM 19286 capable of converting ikarugamycin into butremycin, another PTM [69]. The production of clifednamide B only requires the addition of a hydroxyl group on the macrolactam ring of clifednamide A (Figure S11), which in theory could be performed by the predicted sterol desaturase encoded by *SCLAV_5615* in *S. clavuligerus* (Figure 5B). Further studies are currently underway to elucidate the biosynthesis of clifednamides in *S. clavuligerus.*

### 2.6. Macrolactone Plecomacrolide Antibiotics

**Figure 6:**
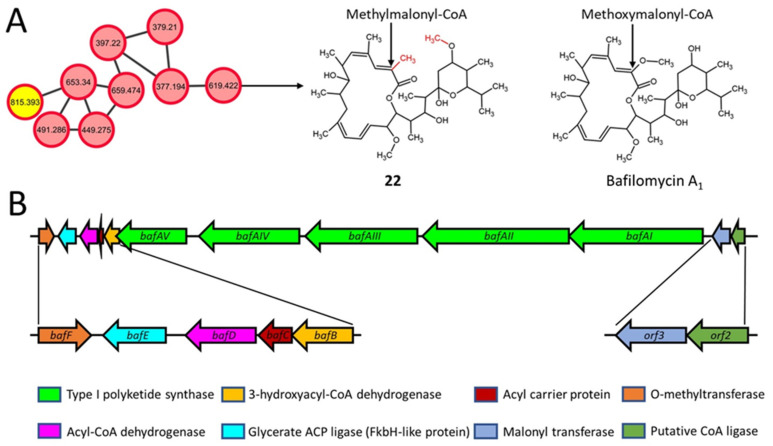
Molecular network (negative ionization mode) and associated biosynthetic gene cluster (BGC) for bafilomycin in *S. clavuligerus*. (**A**) Predicted structure of bafilomycin in a GNPS-based molecular network obtained using network annotation propagation (NAP). Each node depicts a mass spectrum (labeled with *m/z* of the respective precursor mass) and the edges represent the relationship between different nodes. The top-ranked NAP-consensus structural predictions (red boundary) present in all *S. clavuligerus* strains (pink fill) and only in the *rpsL*-K88E overexpression strain (yellow fill) are shown. Bafilomycin A1 is included for comparison with the predicted bafilomycin from the current study. (**B**) The bafilomycin-like BGC present in *S. clavuligerus* is predicted to be involved in producing the metabolite.

Bafilomycins are a group of 16-membered macrolactone plecomacrolide antibiotics produced by various *Streptomyces* species [70,71,72]. Bafilomycin J (**22**, *m/z* 619.422) was detected (consensus rank 2 by NAP) in extracts from wild-type *S. clavuligerus* and the strains overexpressing *frr* and *rpsL* (Figure 6A and Figure S6; Table 2), but not from those expressing the *rpsL* variants. Bafilomycin J is an inhibitor of autophagic protein degradation [71], and a partial bafilomycin-like BGC was identified in *S. clavuligerus* in our analysis (Figure 6B). The BGC contains five type I PKS genes (*bafAI^J^-bafAV^J^*) possessing 12 PKS modules responsible for the formation of the macrolactone ring [70,72]. In the known bafilomycin A_1_ producers *Streptomyces lohii* and *Streptomyces griseus* DSM 2608 (Figure 6A), the macrolactone ring is formed by the thioesterase domain of the *bafAV^A1^* gene product [70,72]. In addition, the acyltransferase (AT) domain of BafAV^A1^ selects methoxymelonyl-CoA for incorporation into bafilomycin A_1_ [70], whereas our analysis suggests that it should be methylmalonyl-CoA in the case of bafilomycin J in *S. clavuligerus* (Figure 6A). It has been reported that the AT domain of BafAV^A1^ from *S. griseus*, which is also conserved in the predicted BafAV^J^ from *S. clavuligerus*, shares a high degree of similarity with AT domains specific for methylmalonyl-CoA [70]. Therefore, it is possible that the use of methylmalonyl-CoA as a precursor could account for the methyl group in bafilomycin J in *S. clavuligerus* as compared to bafilomycin A_1_ (Figure S12). Bafilomycin J also contains a methoxy group on the 6-membered ring instead of the hydroxyl group found on bafilomycin A_1_ (Figure 6A). Based on the proposed pathway leading to bafilomycin J, our results suggest that the *O*-methyltransferase encoded by *bafF* from the *S. clavuligerus* BGC could be responsible for modifying the hydroxyl group in a precursor (Figure S12).

### 2.7. Diterpenoids and Organonitrogen Compounds with no Identifiable BGCs

**Figure 7:**
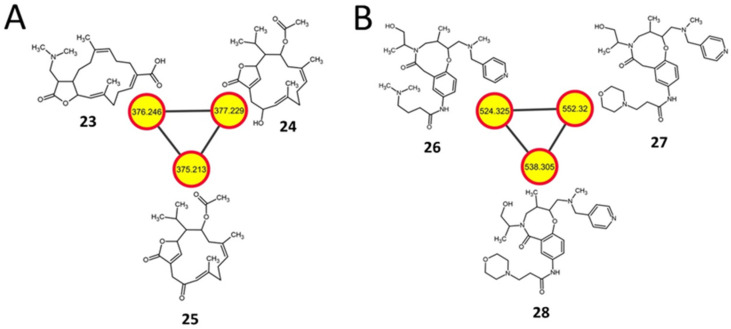
Examples of other molecular networks detected in *S. clavuligerus* strains engineered from the current study. (**A**) A positive ionization mode molecular network of cembrane diterpenoids present in *S. clavuligerus* strains overexpressing different *rpsL* variants (K88E, L90K, R94G). (**B**) A negative ionization mode molecular network of organooxygen and organonitrogen compounds detected in *S. clavuligerus* overexpressing *rpsL* only. The structures were predicted by GNPS-based molecular networking and network annotation propagation (NAP). Each node depicts a mass spectrum (labeled with *m/z* of the respective precursor mass) and the edges represent the relationship between different nodes. The top-ranked NAP-consensus structural predictions (red boundary) present in the overexpression strains (yellow fill) are shown.

Cembrane diterpenoids (cembranolides) are abundant in several genera of soft corals [73] and are also produced by some *Streptomyces* [74]. In the current study, the cembranolides 17-dimethylamino lobohedleolide (**23**, *m/z* 376.246), CID: 11559852 (**24**, *m/z* 377.229) and SN00398992 (**25**, *m/z* 375.213) were detected only in the extracts from the *S. clavuligerus* strains overexpressing the *rpsL* variants (Figure 7A and Figure S7; Table 2). In vitro HIV-inhibitory activity has been reported for **23** [75], whereas to the best of our knowledge, there are no reports on the production or bioactivity of **24** or **25**. There are many terpene-like BGCs present in *S. clavuligerus,* which could be responsible for the biosynthesis of these cembranolides [7,9]. Furthermore, three putative organonitrogen compounds, ChEBI:124407 (**26**, *m/z* 524.325), ChEBI:126491 (**27**, *m/z* 552.32), and ChEBI:128695 (**28**, *m/z* 538.305), were also detected in the same *S. clavuligerus rpsL* extracts (Figure 7B and Figure S8; Table 2). These SMs comprise a heterocyclic ring containing oxygen and nitrogen (Figure 7B) and are derivatives of β-amino acids. Other than their classification, information regarding their biosynthesis or bioactivity is not available. Although the high-intensity peaks between the predicted and experimental spectra of such metabolites did not match, most other smaller peaks did, and the structures were the highest-ranked hits in NAP (Figure S8). The prediction of such uncharacterized putative metabolites in the current study highlights the importance of further examining the metabolic capabilities of well-characterized *Streptomyces* species, including *S clavuligerus*.

## 3. Materials and Methods

### 3.1. Bacterial Strains, Plasmids, Culture Conditions and Molecular Methods

The bacterial strains and plasmids used in the current study are described in Table 1. Unless specified, all media components and reagents were purchased from VWR International, Fisher Scientific, Sigma-Aldrich, or BD Biosciences (Canada). *Escherichia coli* and *S. clavuligerus* strains were cultured and manipulated as described previously [28,76,77]. For metabolite production, *S. clavuligerus* strains were grown on starch asparagine (SA) [77], soy [77], ISP4 [77]; glycerol, sucrose, proline, and glutamic acid (GSPG) [78]; and maltose and yeast extract (MYE) [78] agars.

For the overexpression studies, *frr* and *rpsL* were amplified by PCR (Phusion High-Fidelity PCR Kit, NEB, Canada) using custom oligonucleotide primers with engineered restriction sites (Table 3) and were cloned into pHM11a (Table 1). Fragments containing the respective genes along with the ermE*p (promoter) were released from pHM11a as BamHI-BglII fragments and introduced into the BamHI restriction site of pSET152-*tsr* to prepare the *Streptomyces* overexpression constructs (Table 1). Mutant variants of *rpsL* were prepared by using pSET-*rpsL* (Table 1) as a template, along with engineered primers (Table 3) and the QuikChange Site-Directed Mutagenesis Kit, as per the manufacturer’s instructions (Agilent, USA). All oligonucleotide primers used in the current study for PCR amplification and DNA manipulation are listed in Table 3. The DNA sequences of all PCR products and plasmid inserts were determined and confirmed at the Centre for Applied Genomics, University of Toronto, Canada. Plasmids were introduced into *S. clavuligerus* by intergeneric conjugation to prepare the respective overexpression strains (Table 1) [28,77].

### 3.2. Metabolite Extraction, LC-MS/MS Analysis, and Molecular Networking

Each strain of *S. clavuligerus* (Table 1) was grown on solid agar media at 28 °C for seven days to prepare the methanol and ethyl acetate extracts (5 mL), as described previously [7]. The supernatants were filtered, dried in vacuo at 40 °C, and redissolved in 1 mL of the respective solvent (methanol or ethyl acetate). One hundred microliters of the final extracts with 0.2 µM of amitriptyline (internal standard) added were transferred to a 96-well plate for untargeted LC-MS/MS analysis. A Vanquish ultra-high performance liquid chromatography (UHPLC) system-coupled Q Exactive Hybrid Quadrupole-Orbitrap Mass Spectrometer (Thermo Scientific, United States) was used to analyze the samples. Chromatographic separation was performed in mixed mode (allowing weak anion/cation exchange) on a Scherzo SM-C18 column (2 × 250 mm, 3 μm, 130 Å; Imtakt, United States) maintained at 40 °C. Chromatography was performed with 10 µL of each sample at a flow rate of 0.5 mL/min with a mobile phase consisting of (A) 0.1% formic acid in water and (B) 0.1% formic acid in acetonitrile. The following program was used: 0–5 min, 98% A; 5–8 min, gradient of 98–50% A (or 50% B); 8–13 min, gradient 50–100% B; 13–14.00 min, 100% B; 14–14.10 min, 100–2% B; 14.10–18 min, 2% B.

A heated electrospray ionization source with a heater capillary temperatures of 370 °C and 350 °C, respectively, was used to perform the mass spectrometry in either positive or negative ionization mode, with the following parameters: ± 3000.0 V; S-lens RF, 55; sheath gas flow rate, 55; and auxiliary gas flow rate, 20. MS1 and MS2 scans (at 200 *m/z*) were acquired from 0.48 to 16.0 min for the 100–1500 *m/z* range at resolutions of 35,000 and 17,500, respectively. The maximum injection time and automatic gain control target values were set at 150 ms and 5 × 10^5^. Up to four MS2 scans in the data-dependent mode were acquired for the most abundant ions per duty cycle, with a starting value of 70 *m/z* and an exclusion parameter of 10 s. Higher-energy collision-induced dissociation was performed with normalized collision energies of 20, 35, and 50 eV. The apex trigger mode was used (2–7 s), and the isotopes were excluded [7]. The obtained raw LC-MS/MS data files were converted to. mzXML format in ProteoWizard [79], with 32-bit binary encoding precision, zlib compression, and peak peaking. Classical molecular networks were generated separately for the positive and negative ionization modes with GNPS, using listed parameters (Table S1) [26]. The resulting networks (and all others) were visualized and interpreted in Cytoscape 3.8 [80], and the nodes corresponding to uninoculated media (control) and the duplicated nodes with the same *m/z* (if part of the same molecular network) were removed manually. Metabolites were annotated in GNPS by spectral matching of fragmentation spectra with those present in public spectral libraries [26]. Spectra were validated manually using mirror plots (maximum ion mass accuracy = 5 ppm) corresponding to level 2 annotation based on the Minimum Standard Initiative [81]. All the metabolomics data is publicly available (https://massive.ucsd.edu/ProteoSAFe/dataset.jsp?accession=MSV000085619, uploaded on 22 June 2020).

### 3.3. In Silico Annotation of the Metabolites

Network annotation propagation (NAP) [27,82] in GNPS was used to provide in silico structural annotation. NAP was run using the standard “NAP_CCMS” workflow for both the positive ([M+H]^+^, [M+Na]^+^, [M+NH_4_]^+^, and [M+K]^+^) and negative ([M–H]^–^) ionization modes, using the recommended parameters (Table S1). The GNPS, Human Metabolome (HMDB), Super Natural II (SUPNAT), Chemical Entities of Biological Interest (ChEBI), DRUGBANK, and FooDB structural databases were used in NAP. The predicted structures of the metabolites in the molecular networks were visualized in Cytoscape using the chemViz2 plugin [27]. The candidate structure from the NAP consensus score was used for analysis and reporting. The metabolites discussed in the current study were selected based on either their presence in only the overexpression strains or if they could be matched as a product of a putative gene cluster from *S. clavuligerus*. For each putative metabolite, spectral matching of the predicted (by NAP/MetFrag) and experimental (by LC-MS/MS) fragmentation spectra was conducted using MetFrag [82] to further validate the annotation (maximum ion mass accuracy = 5 ppm). In some cases, SIRIUS [83] was also used to validate the predicted structures. The annotated spectra were added to the GNPS spectral library (CCMSLIB00005788068-90).

## 4. Conclusions

This is the first study to examine the effect of overexpressing *frr*, *rpsL*, and *rpsL* variants on specialized metabolism in *S. clavuligerus* using untargeted metabolomics, GNPS-based molecular networking, and in silico metabolite annotation. We putatively annotated 28 metabolites from eight different classes, which have not been previously reported in *S. clavuligerus*. Other studies have shown that the manipulation of *frr* and *rpsL* increases the production of known SMs in some *Streptomyces* spp. [13]. In addition, the introduction of the *rpsL*-K88E mutation has been reported to promote the expression of *frr* through an unknown mechanism [17], suggesting a possible mechanistic link leading to overlapping phenotypic outcomes. In the current study, it was found that certain metabolites were specifically detected on either *frr* or *rpsL*-variant overexpression in *S. clavuligerus*, suggesting that the two mechanisms can sometimes function independently. Overexpression of either *frr* or *rpsL*-K88E is known to enhance protein synthesis in other *Streptomyces* spp. [15,24], and the latter also leads to the formation of more stable 70S ribosome complexes [15]. The reasons for enhanced SM production in other *rpsL* variants are still unclear but are thought to involve a somewhat separate mechanism as compared to *rpsL*-K88E [21]. Therefore, it is possible that differential perturbations in ribosome function due to *frr* or *rpsL*-variant overexpression in *S. clavuligerus* somehow leads to altered SM production profiles. Furthermore, we demonstrate that the synthesis of putative plant-associated metabolites, including triterpenoids, triterpenoid glycosides, and flavonoid glycosides, can be promoted under laboratory conditions in *S. clavuligerus* through ribosome manipulation. Other putative SMs such as streptovirudins, PTMs, and bafilomycin J are also reported for the first time in *S. clavuligerus*. In addition, we matched *S. clavuligerus* BGCs with some of the predicted SMs, and propose biosynthetic routes based on similar systems. The combined application of GNPS and NAP not only predicted the discussed structures but also pointed to novel SMs such as one (*m/z* 843.423) from the molecular network of streptovirudins. It should be noted that further investigations are warranted to elucidate some of the described findings. Overall, our results suggest that industrial microorganisms such as *S. clavuligerus* harbor the potential to produce additional metabolites under laboratory conditions and that the current approach employing molecular networking and in silico annotation can be used to hasten their discovery.

## Figures and Tables

**Table 1:** Bacterial strains and plasmids used in the current study.

Strain/Plasmid	Description ^a^	Source/Reference ^b^
**Bacterial Strains**		
*Escherichia coli* DH5α	General laboratory cloning host	Promega
*E. coli* ET12567/pUZ8002	DNA methylation deficient conjugation host containing the plasmid pUZ8002 (Cam ^R^, Kan ^R^)	[28]
*Streptomyces clavuligerus* ATCC 27064	Wild type clavulanic acid producer	ATCC
S. clavuligerus ermE*p	*S. clavuligerus* harboring pSET-ermE*p, control strain	This study
*S. clavuligerus frr*	*S. clavuligerus* harboring pSET*-frr,* overexpression of *frr*	This study
*S. clavuligerus rpsL*	*S. clavuligerus* harboring pSET*-rpsL,* overexpression of *rpsL*	This study
*S. clavuligerus rpsL-*K88E	*S. clavuligerus* harboring pSET*-rpsL-*K88E, overexpression of *rpsL-*K88E	This study
*S. clavuligerus rpsL-*L90K	*S. clavuligerus* harboring pSET*-rpsL-*L90K, overexpression of *rpsL-*L90K	This study
*S. clavuligerus rpsL-*R94G	*S. clavuligerus* harboring pSET*-rpsL-*R94G, overexpression of *rpsL-*R94G	This study
**Plasmids**		
pHM11a	Integrative *Streptomyces* expression vector containing the constitutive ermE*p (Hyg ^R^)	[29]
pSET152-*tsr*	Integrative *Streptomyces* cloning vector (Apr ^R^, Tsr ^R^)	[30,31]
pSET-ermE*p	pSET152-*tsr* containing constitutive promoter ermE*p from pHM11a	This study
pSET-*frr*	pSET152 containing *S. clavuligerus frr* gene along with ermE*p from pHM11a	This study
pSET-*rpsL*	pSET152 containing *S. clavuligerus rpsL* gene along with ermE*p from pHM11a	This study
pSET-*rpsL*-K88E	A site-directed mutant of *rpsL* (Lys88Glu, K88E) in pSET-*rpsL* plasmid	This study
pSET-*rpsL*-L90K	A site-directed mutant of *rpsL* (Leu90Lys, L90K) in pSET-*rpsL* plasmid	This study
pSET-*rpsL*-R94G	A site-directed mutant of *rpsL* (Arg94Gly, R94G) in pSET-*rpsL* plasmid	This study

^a^ Cam ^R^, chloramphenicol resistance; Kan ^R^, kanamycin resistance, Hyg ^R^, hygromycin resistance; Apr ^R^, apramycin resistance; Tsr ^R^, thiostrepton resistance. ^b^ ATCC, American Type Culture Collection.

**Table 2:** In silico annotation of metabolites produced by wild-type (wt) *S. clavuligerus* or strains overexpressing *frr* (frr), *rpsL* (rpsL), *rpsL*-K88E (K88E), *rpsL*-L90K (L90K) and *rpsL*-R94G (R94G).

Label	Observed *m/z* [Adduct]	Name/ Database ID ^a^	Strain Detected in	Molecular Formula (Weight, g/mol)	Metabolite Family
1	1497.64 [M−H]^−^	Securioside A	K88E	C_72_H_106_O_33_ (1499.6)	Triterpenoid glycoside
2	1455.63 [M−H]^−^	CID: 56924794	K88E	C_70_H_104_O_32_ (1457.6)	Triterpenoid glycoside
3	1057.52 [M−H]^−^	Eryngioside E	K88E	C_52_H_82_O_22_ (1059.2)	Triterpenoid glycoside
4	1087.53 [M−H]^−^	CID: 10582011	K88E, L90K	C_53_H_84_O_23_ (1089.2)	Triterpenoid glycoside
5	1073.52 [M−H]^−^	Tragopogonsaponin Q	K88E, L90K	C_56_H_82_O_20_ (1075.2)	Triterpenoid glycoside
6	1101.51 [M−H]^−^	SN00394245	K88E	C_53_H_82_O_24_ (1103.2)	Triterpenoid glycoside
7	1103.53 [M−H]^−^	CID: 101205416	K88E, L90K	C_53_H_84_O_24_ (1105.2)	Triterpenoid glycoside
8	750.31 [M−H]^−^	Mannooligosaccharide derivative	K88E, L90K	C_29_H_53_NO_21_ (751.3)	Saccharide
9	441.372 [M+H]^+^	22-Hydroxy-2-hopen-1-one^b^	K88E, L90K, R94G	C_30_H_48_O_2_ (440.7)	Hopanoid
10	439.357 [M−H_2_O+H]^+^	Oleanolic acid^b^	K88E	C_30_H_48_O_3_ (456.7)	Triterpenoid
11	423.362 [M+H]^+^	Glochidone	K88E, L90K, R94G	C_30_H_46_O (422.3)	Hopanoid
12	617.404 [M+H]^+^	CID: 10603865	K88E	C_36_H_56_O_8_ (616.8)	Triterpenoid glycoside
13	599.393 [M+H]^+^	SN00379882	K88E, L90K, R94G	C_36_H_54_O_7_ (598.4)	Triterpenoid
14	727.218 [M−H]^−^	Viscumneoside V	K88E	C_32_H_40_O_19_ (728.2)	Flavonoid glycoside
15	681.212 [M−H]^−^	CID: 42607862	K88E	C_31_H_38_O_17_ (682.6)	Flavonoid glycoside
16	741.233 [M−H]^−^	Monoglucosyl naringin	K88E	C_33_H_42_O_19_ (742.7)	Flavonoid glycoside
17	819.42 [M+H]^+^	Streptovirudin C1	frr	C_37_H_62_N_4_O_16_ (818.9)	Nucleoside antibiotic
18	791.393 [M+H]^+^	Streptovirudin A1	frr	C_35_H_58_N_4_O_16_ (790.9)	Nucleoside antibiotic
19	511.28 [M+H]^+^	Maltophilin	wt, frr, rpsL, K88E, L90K, R94G	C_29_H_38_N_2_O_6_ (510.6)	Polycyclic tetramate macrolactam
20	509.264 [M+H]^+^	Clifednamide B	wt, frr, rpsL, K88E, L90K, R94G	C_29_H_36_N_2_O_6_ (508.6)	Polycyclic tetramate macrolactam
21	493.27 [M+H]^+^	Clifednamide A	K88E, L90K, R94G	C_29_H_36_N_2_O_5_ (492.6)	Polycyclic tetramate macrolactam
22	619.422 [M-H]^−^	Bafilomycin J	wt, frr, rpsL	C_36_H_60_O_8_ (620.9)	Macrolide
23	376.246 [M+H]^+^	17-dimethylamino lobohedleolide	K88E	C_22_H_33_NO_4_ (375.2)	Cembrane diterpenoid
24	377.229 [M+H]^+^	CID: 11559852	K88E, L90K, R94G	C_22_H_32_O_5_ (376.5)	Cembrane diterpenoid
25	375.213 [M+H]^+^	SN00398992	K88E	C_22_H_30_O_5_ (374.2)	Cembrane diterpenoid
26	524.325 [M−H]^−^	ChEBI:124407	rpsL	C_29_H_43_N_5_O_4_ (525.3)	Organonitrogen
27	552.32 [M−H]^−^	ChEBI:126491	rpsL	C_30_H_43_N_5_O_5_ (553.3)	Organonitrogen
28	538.305 [M−H]^−^	ChEBI:128695	rpsL	C_29_H_41_N_5_O_5_ (539.3)	Organonitrogen

^a^ Database ID: CID (PubChem, https://pubchem.ncbi.nlm.nih.gov/), SN (Super Natural II, http://bioinf-applied.charite.de/supernatural_new/index.php?site=home), and ChEBI (Chemical Entities of Biological Interest, https://www.ebi.ac.uk/chebi/). All databases were accessed on or before 31 January 2021. ^b^ Both 22-Hydroxy-2-hopen-1-one and oleanolic acid were detected by GNPS.

**Table 3:** Sequences of the oligonucleotide primers used in the current study and their details.

Primer Name	Sequence (5’→3’)	Description
Sc-frr-F	ATAGCCATATGATGGAGAAGGCCGTCGTGGTC	Primers for the amplification of *frr* from *S. clavuligerus* to prepare pSET-*frr*
Sc-frr-R	CTTACGGATCCTCAGACTTCGAGCAGCTCGG	
Sc-rpsL-F	ATAGCCATATGGTGCCTACGATCCAGCAGC	Primers for the amplification of *rpsL from S. clavuligerus* to prepare pSET-*rpsL*
Sc-rpsL-R	CTTACGGATCCTTACTTCTCCTTCTTGGCG	
Sc-rpsL-K88E-F	CCGGCAGGTCCTCCACACGGCCACC	Primers for introducing a single amino acid mutation (Lys88Glu) in *rpsL* from *S. clavuligerus* to prepare pSET-*rpsL*-K88E
Sc-rpsL-K88E-R	GGTGGCCGTGTGGAGGACCTGCCGG	
Sc-rpsL-L90K-F	TAACGAACACCCGGCTTGTCCTTCACACGGCC	Primers for introducing a single amino acid mutation (Leu90Lys) in *rpsL* from *S. clavuligerus* to prepare pSET-*rpsL*-L90K
Sc-rpsL-L90K-R	GGCCGTGTGAAGGACAAGCCGGGTGTTCGTTA	
Sc-rpsL-R94G-F	CGGATGATCTTGTAACCAACACCCGGCAGGTC	Primers for introducing a single amino acid mutation (Arg94Gly) in *rpsL* from *S. clavuligerus* to prepare pSET-*rpsL*-R94G
Sc-rpsL-R94G-R	GACCTGCCGGGTGTTGGTTACAAGATCATCCG	

## Data Availability

All data relevant to the current study have been submitted to public databases and is freely available. GNPS positive mode: https://gnps.ucsd.edu/ProteoSAFe/status.jsp?task=6644bda70eca4c6fa186451c816435f8. GNPS negative mode: https://gnps.ucsd.edu/ProteoSAFe/status.jsp?task=1d86e5bcaa7048419a80c6849ee4fb93. NAP positive mode: https://proteomics2.ucsd.edu/ProteoSAFe/status.jsp?task=6c0362f5388a42d296f0b6eeaf54878b. NAP negative mode: https://proteomics2.ucsd.edu/ProteoSAFe/status.jsp?task=febaa77b47ca42debb328aaec5c8dadc. Original data: https://massive.ucsd.edu/ProteoSAFe/dataset.jsp?accession=MSV000085619.

## Supplementary Materials

The following are available online at https://www.mdpi.com/article/10.3390/metabo11040239/s1. Figure S1: Comparison plots showing mass spectrometry fragmentation patterns of putative metabolites (1–8) from the first triterpenoid molecular network. Figure S2: Comparison plots showing mass spectrometry fragmentation patterns of putative metabolites (9–13) from the second triterpenoid (hopanoid) molecular network. Figure S3: Comparison plots showing mass spectrometry fragmentation patterns of putative metabolites (14–16) from the flavonoid molecular network. Figure S4: Comparison plots showing mass spectrometry fragmentation patterns of putative metabolites (17–18) from the nucleoside antibiotic molecular network, Figure S5: Comparison plots showing mass spectrometry fragmentation patterns of putative metabolites (19–21) from the polycyclic tetramate macrolactam molecular network. Figure S6: Comparison plots showing the predicted and experimental mass spectrometry fragmentation pattern of bafilomycin J (22). Figure S7: Comparison plots showing mass spectrometry fragmentation patterns of putative diterpenoid metabolites (23–25) from the current study. Figure S8: Comparison plots showing mass spectrometry fragmentation patterns of putative organonitrogen metabolites (26–28) from the current study. Figure S9: The mevalonic acid-based plant triterpene biosynthetic pathway. Figure S10: The proposed biosynthetic pathway for flavonoids and derived glycosides in S. clavuligerus. In the scheme leading to ferulic acid in *S. clavuligerus*, 4-coumarate-3-hydroxylase and caffeic acid O-methyltransferase are predicted to be encoded by *ncyP* and *SCLAV_5485*, respectively (shown in red), as their predicted gene products contain the required protein domains. In the current study, caffeic acid was also detected by GNPS in some of the *S. clavuligerus* overexpression strains (including rpsL-K88E). Figure S11: The proposed biosynthetic scheme for clifednamide A and B from ikarugamycin in *S. clavuligerus*. Figure S12: The proposed biosynthetic scheme for bafilomycin J involving putative products from a partial gene cluster present in *S. clavuligerus*. Table S1: Global natural products social molecular networking (GNPS) and network annotation propagation (NAP) parameters used for analysis in the current study.
