# Supplementary material for: Specialized Metabolites from Ribosome Engineered Strains of Streptomyces clavuligerus
Source: Metabolites. 2021 Apr 13;11(4):239. doi: 10.3390/metabo11040239 (PMC8069389; doi:10.3390/metabo11040239)
Supplement: Supplementary file 1 [file metabolites-11-00239-s001.pdf]

Figure S1: Comparison plots showing mass spectrometry fragmentation patterns of putative metabolites (1-8) from the first triterpenoid molecular network. Peaks shared between predicted and experimental spectra are indicated in green and those not matched are in blue.

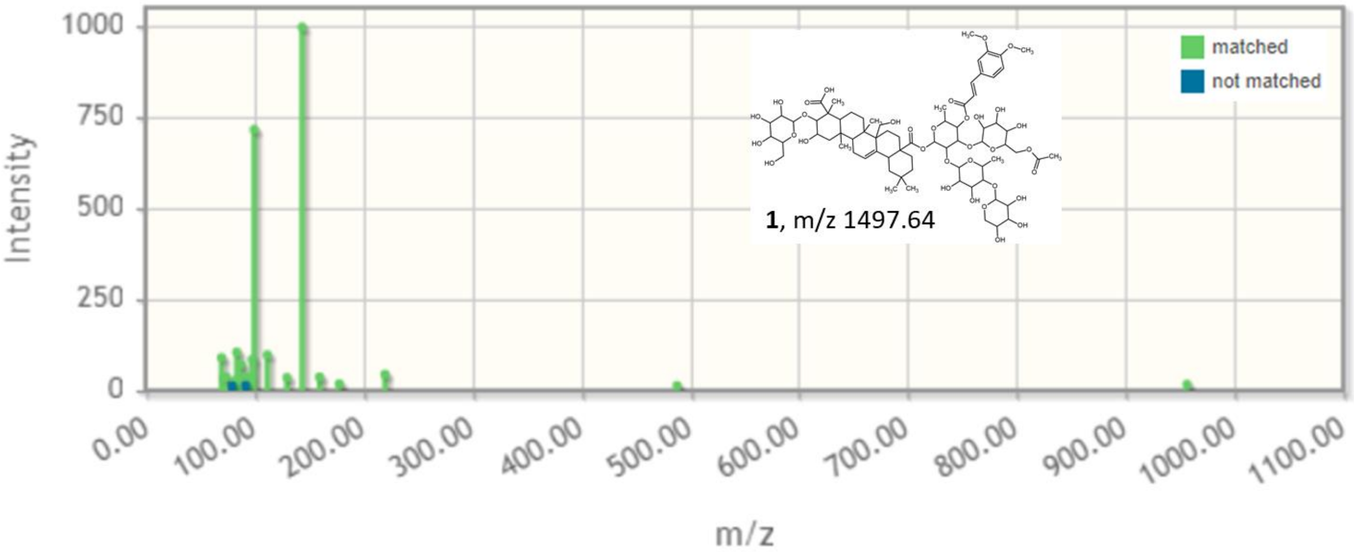

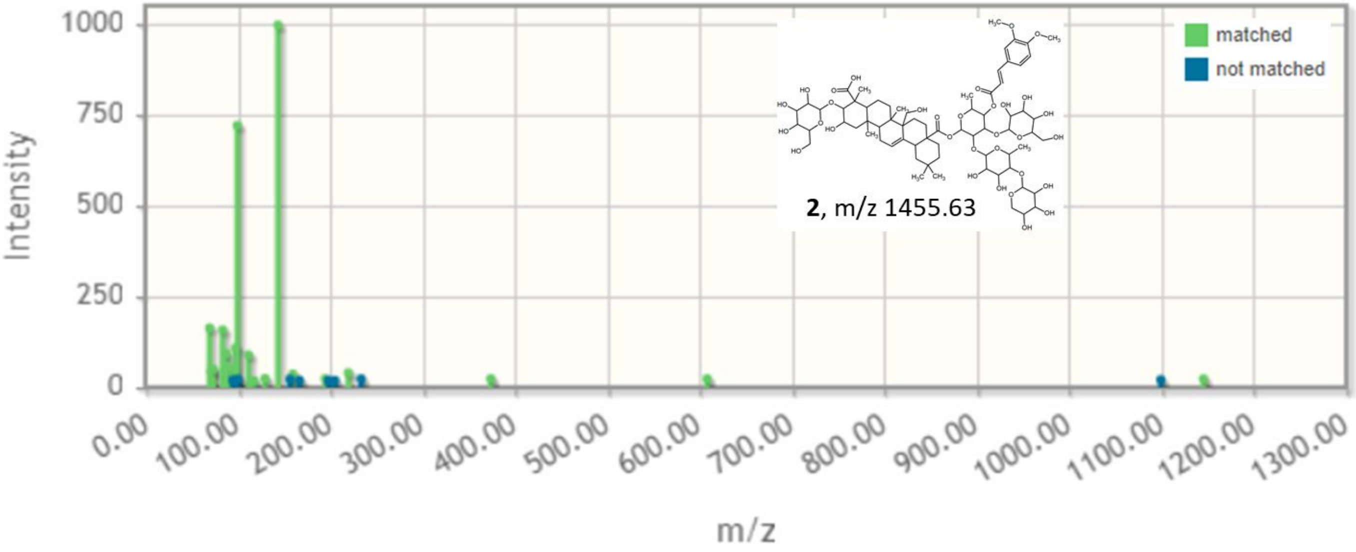

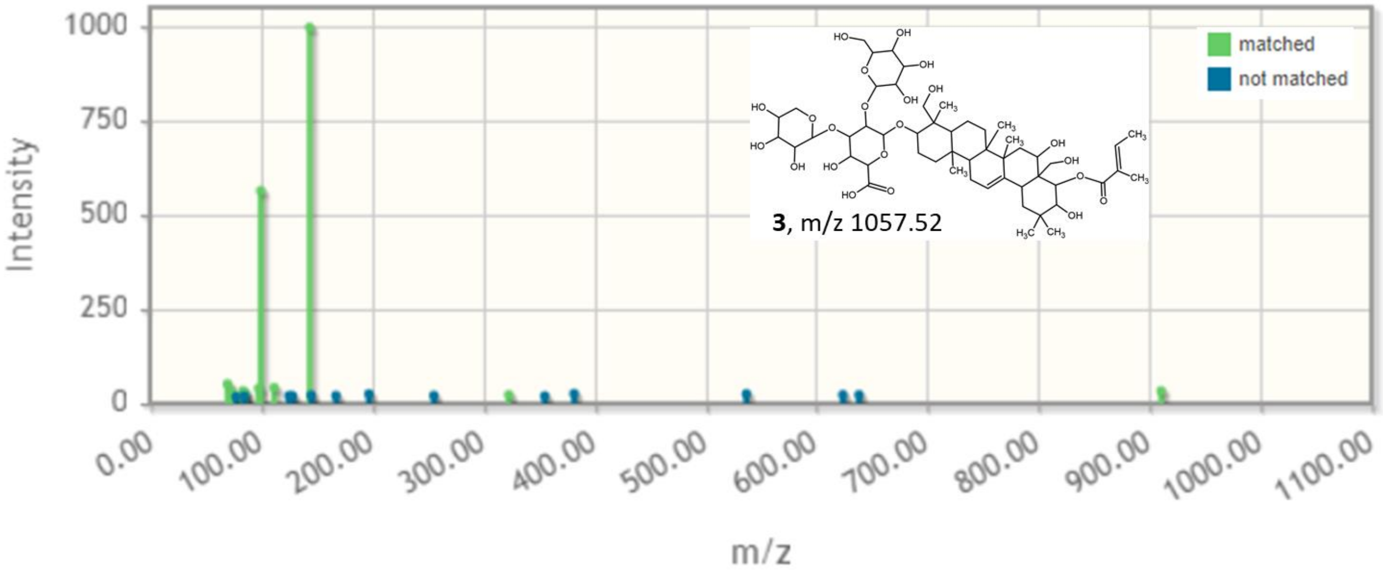

1000
750
500
250
0
Intensity
0.00
100.00
200.00
300.00
400.00
500.00
600.00
700.00
800.00
900.00
1000.00
1100.00
m/z
matched
not matched
3, m/z 1057.52

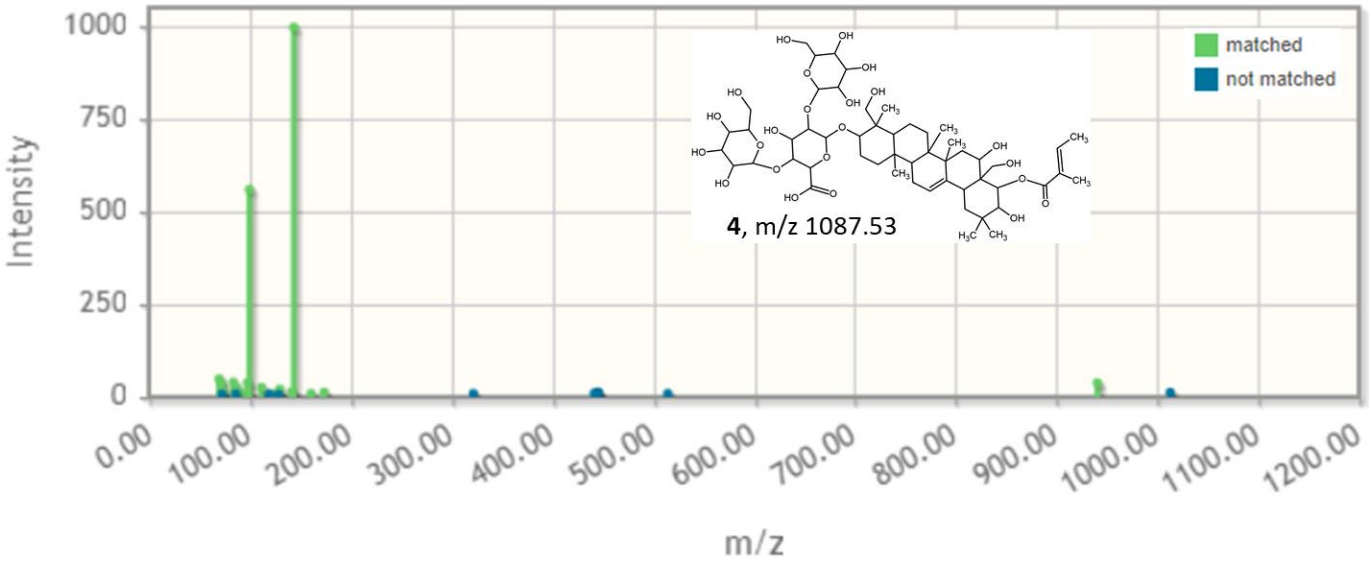

1000
750
500
250
0
Intensity
0.00
100.00
200.00
300.00
400.00
500.00
600.00
700.00
800.00
900.00
1000.00
1100.00
1200.00
m/z
matched
not matched
4, m/z 1087.53

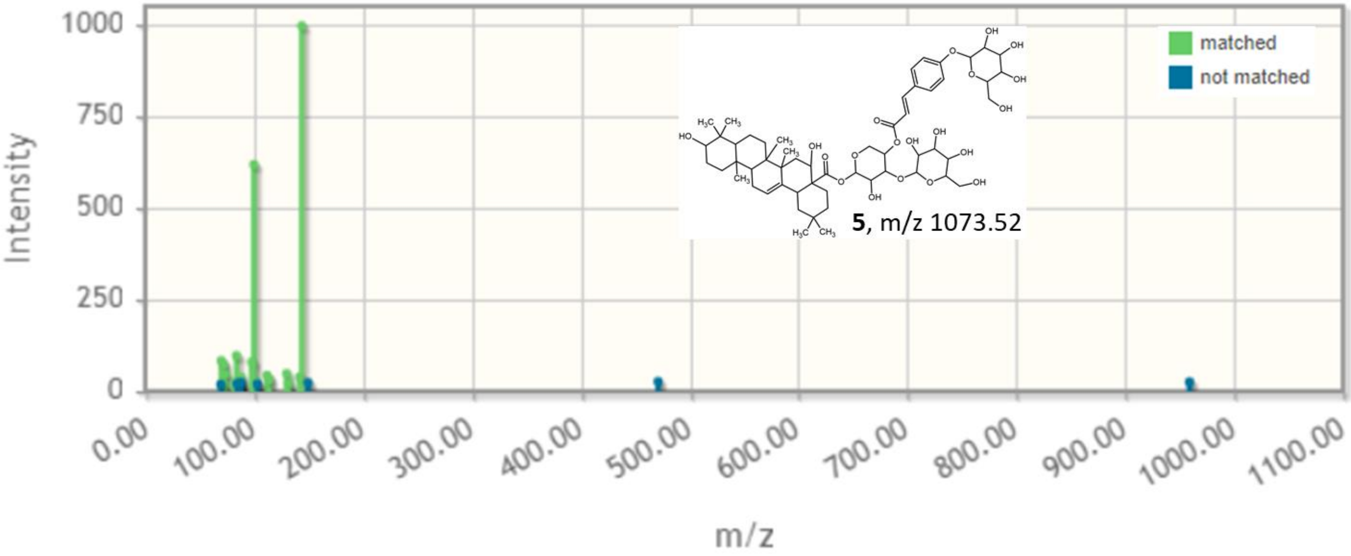
1000
750
500
250
0
Intensity
0.00
100.00
200.00
300.00
400.00
500.00
600.00
700.00
800.00
900.00
1000.00
1100.00
m/z
matched
not matched
5, m/z 1073.52

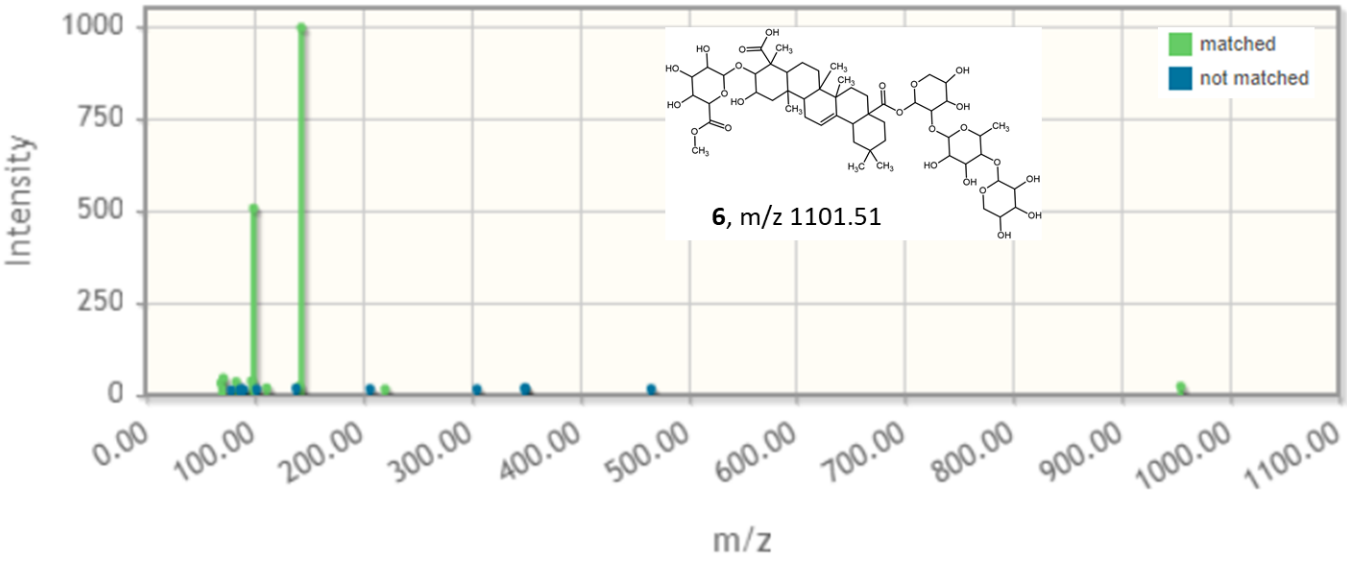
1000
750
500
250
0
Intensity
0.00
100.00
200.00
300.00
400.00
500.00
600.00
700.00
800.00
900.00
1000.00
1100.00
m/z
matched
not matched
6, m/z 1101.51

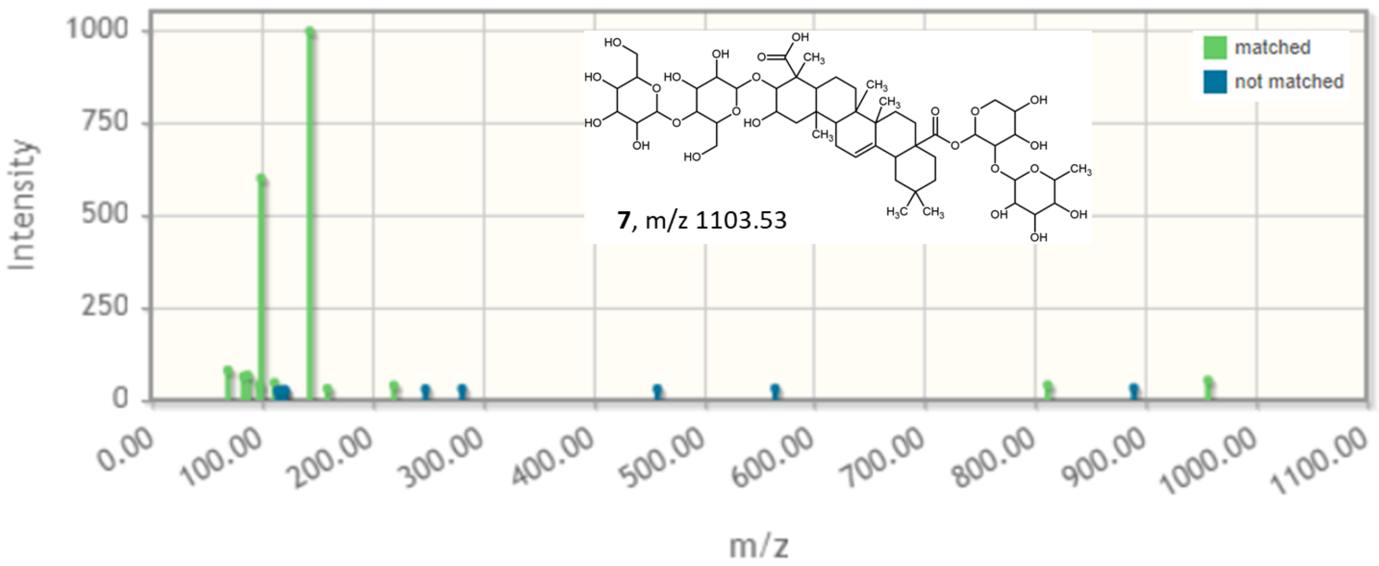

Intensity
1000
750
500
250
0
matched
not matched
7, m/z 1103.53
0.00
100.00
200.00
300.00
400.00
500.00
600.00
700.00
800.00
900.00
1000.00
1100.00
m/z

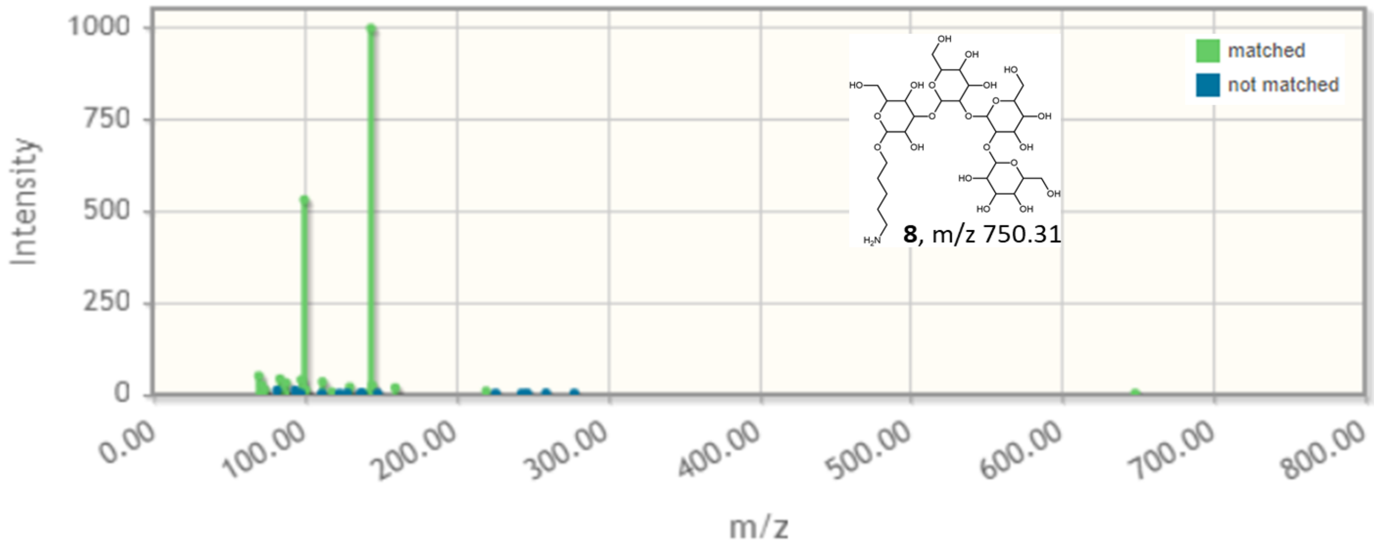

Intensity
1000
750
500
250
0
matched
not matched
8, m/z 750.31
0.00
100.00
200.00
300.00
400.00
500.00
600.00
700.00
800.00
m/z

Figure S2: Comparison plots showing mass spectrometry fragmentation patterns of putative metabolites (9-13) from the second triterpenoid (hopanoid) molecular network. Peaks shared between predicted and experimental spectra are indicated in green and those not matched are in blue.

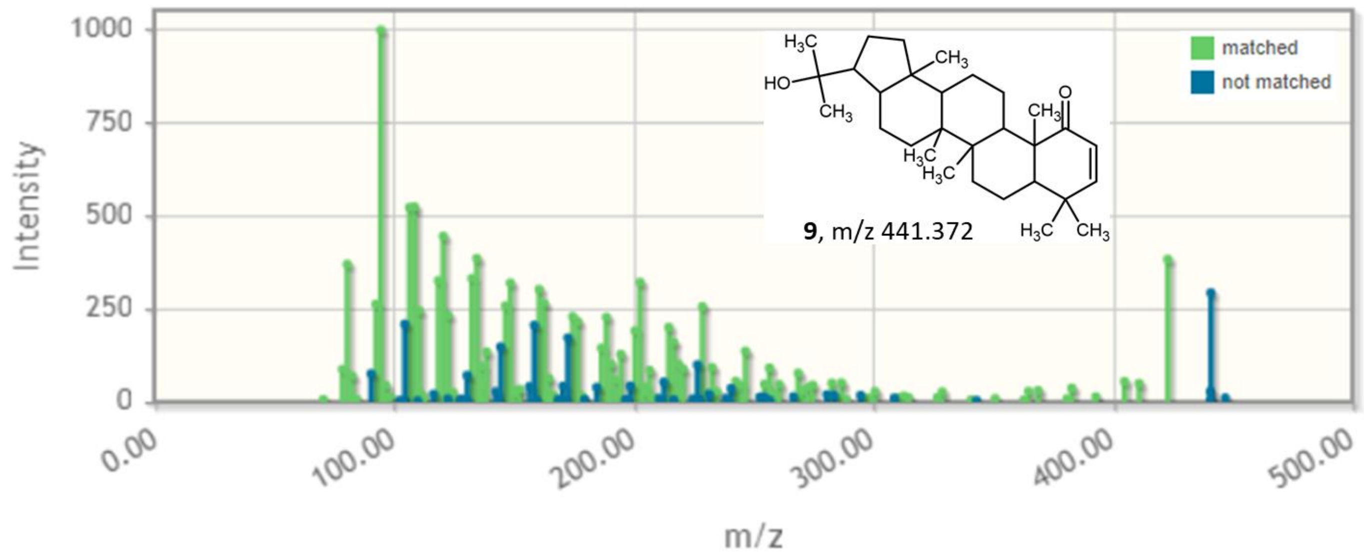

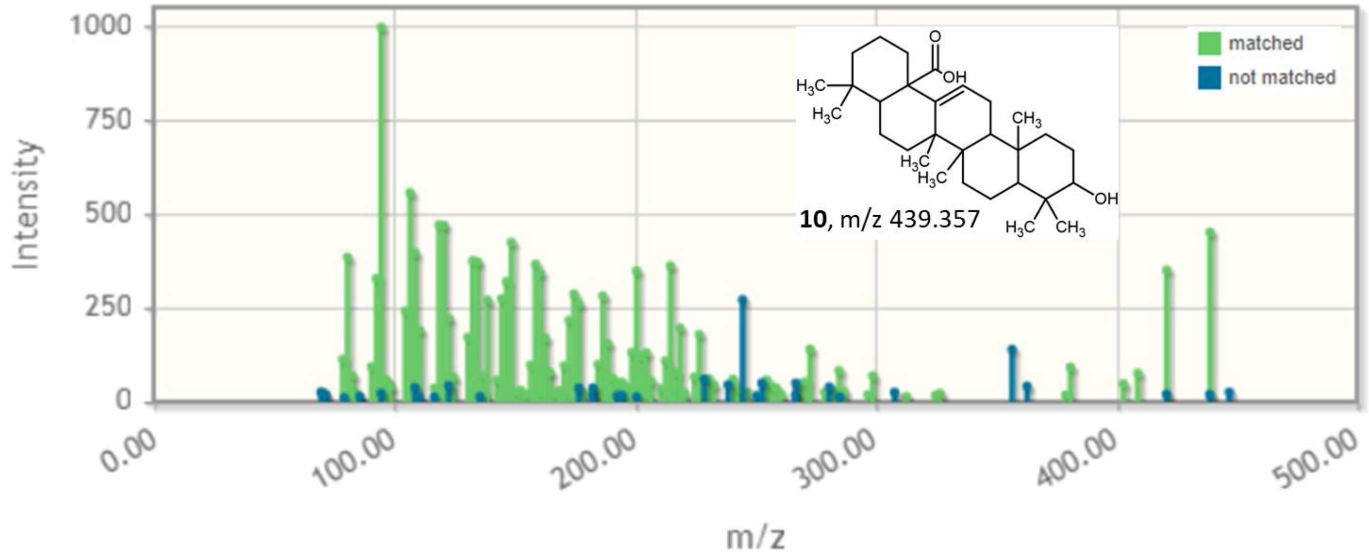

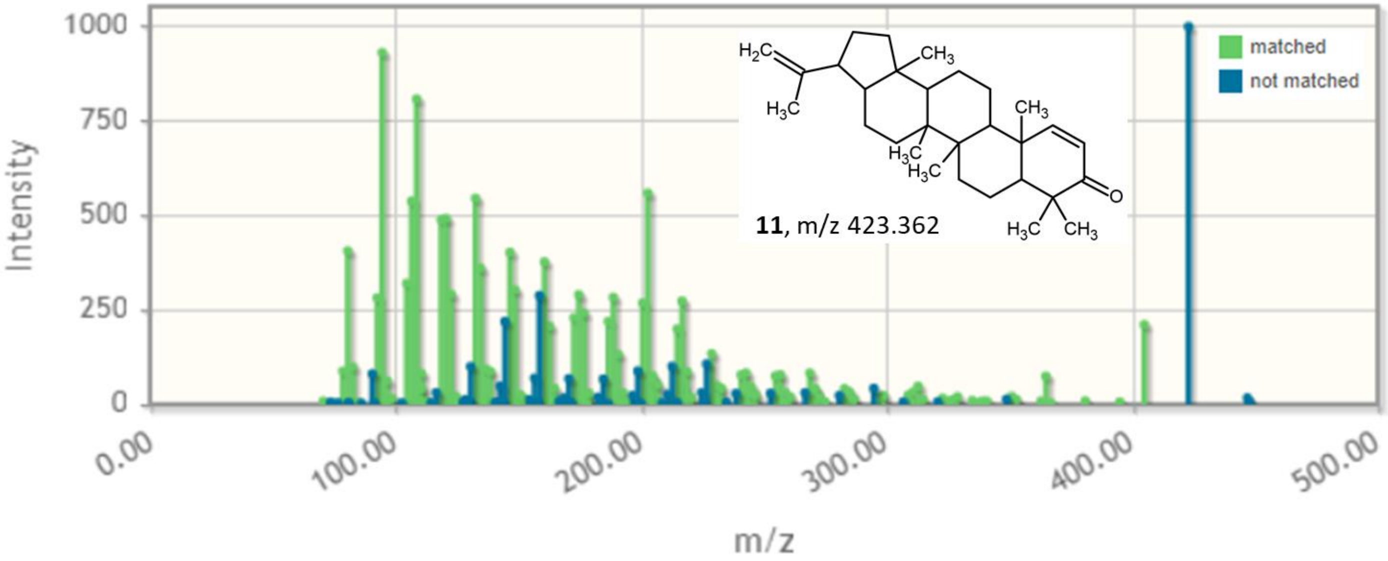

Intensity
m/z
matched
not matched
11, m/z 423.362

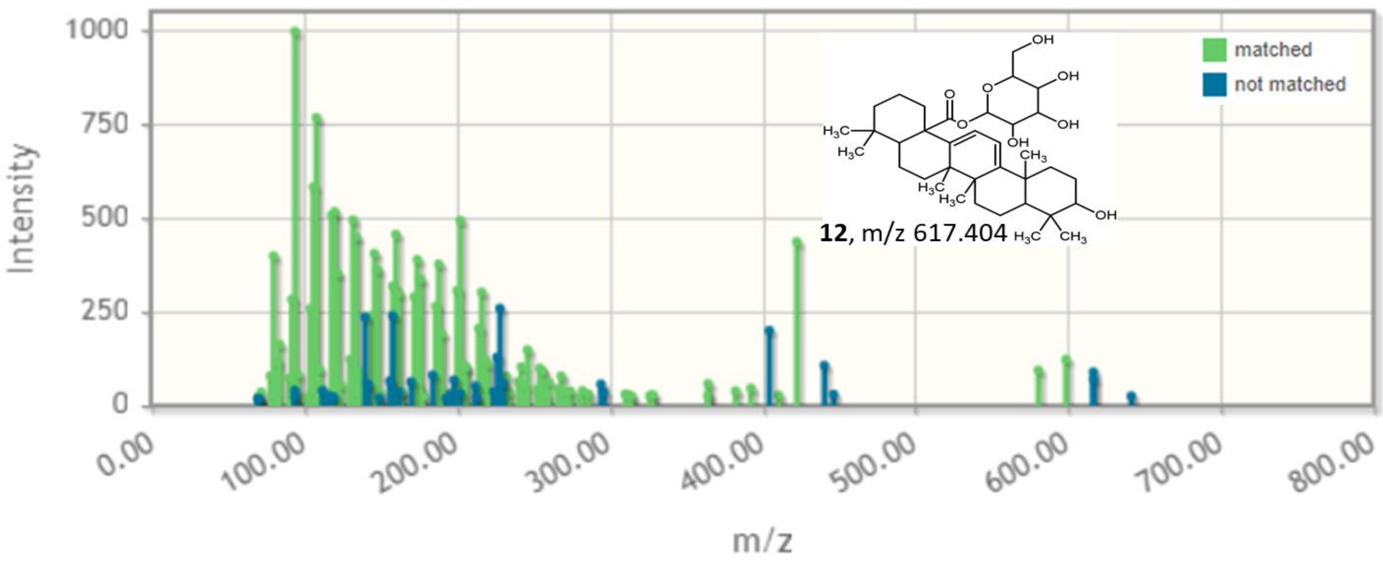

Intensity
m/z
matched
not matched
12, m/z 617.404

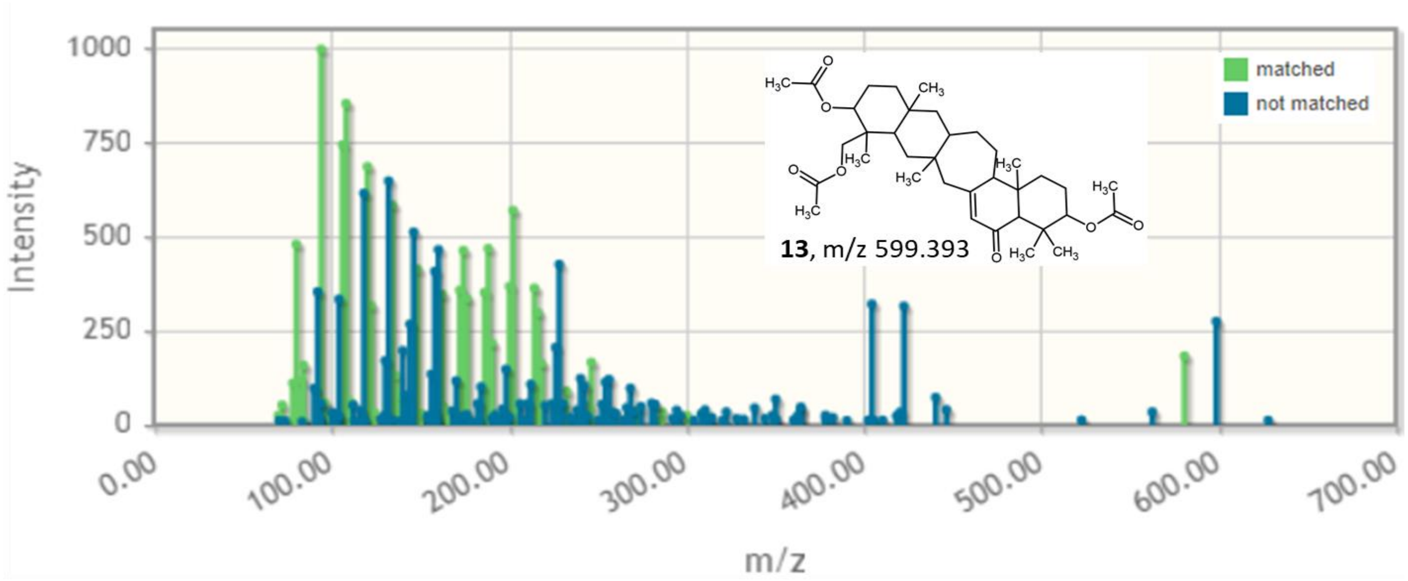

1000
750
500
250
0
Intensity
0.00
100.00
200.00
300.00
400.00
500.00
600.00
700.00
m/z
matched
not matched
13, m/z 599.393

Figure S3: Comparison plots showing mass spectrometry fragmentation patterns of putative metabolites (14-16) from the flavonoid molecular network. Peaks shared between predicted and experimental spectra are indicated in green and those not matched are in blue.

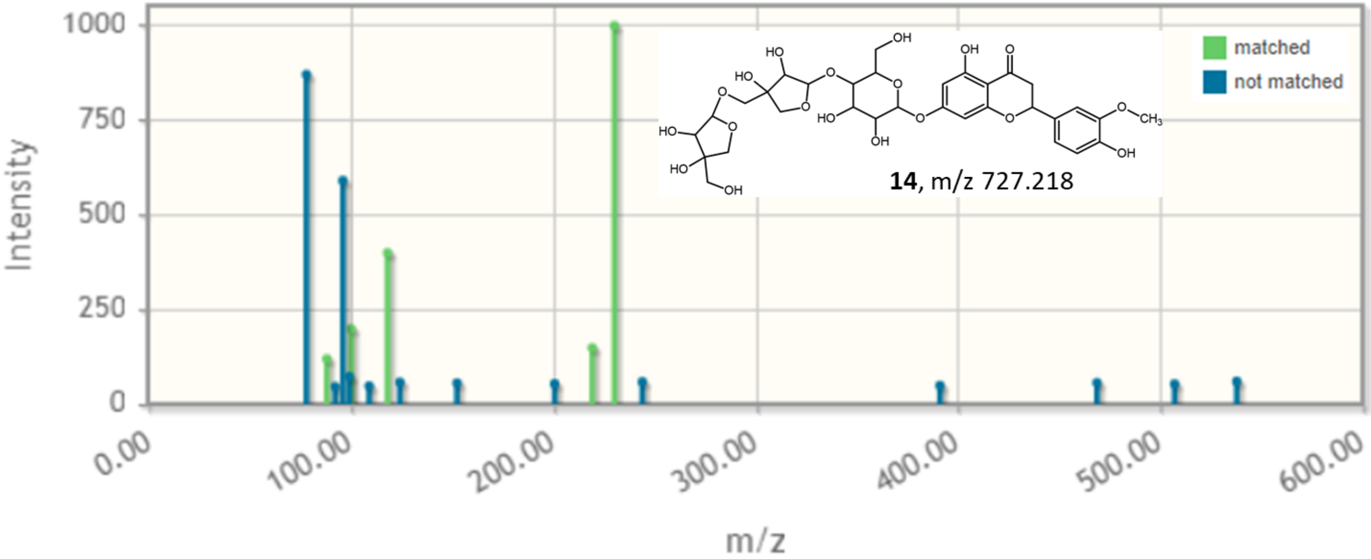

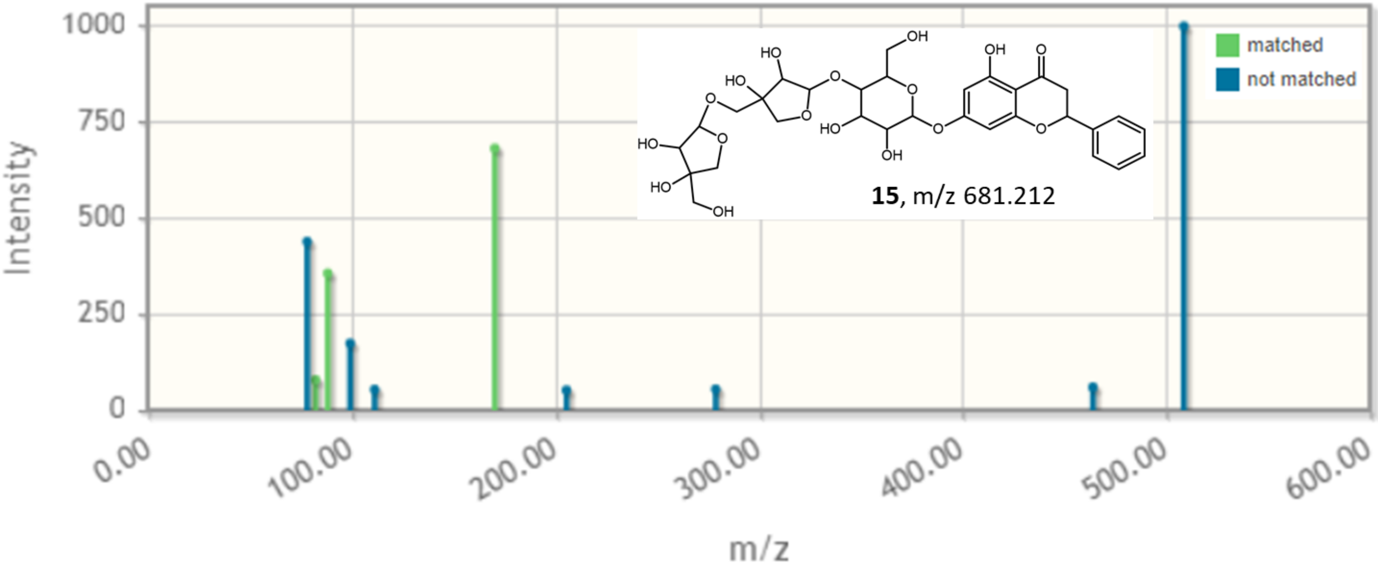

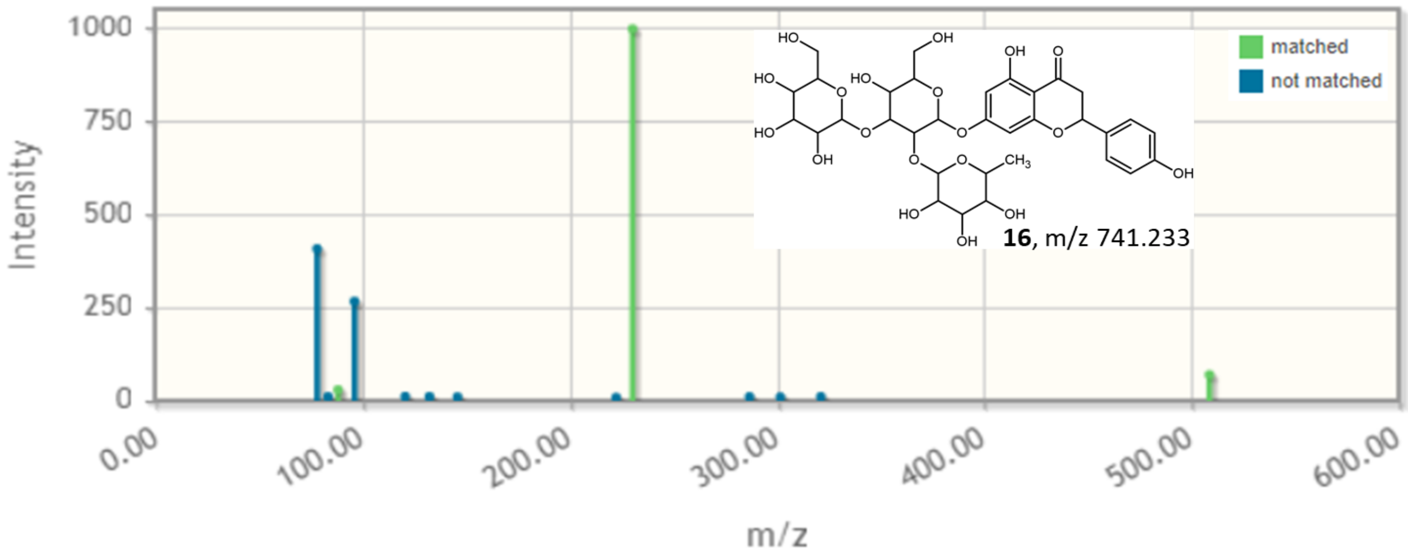
1000
750
500
250
0
Intensity
0.00
100.00
200.00
300.00
400.00
500.00
600.00
m/z
matched
not matched
16, m/z 741.233

Figure S4: Comparison plots showing mass spectrometry fragmentation patterns of putative metabolites (17-18) from the nucleoside antibiotic molecular network. Peaks shared between predicted and experimental spectra are indicated in green and those not matched are in blue.

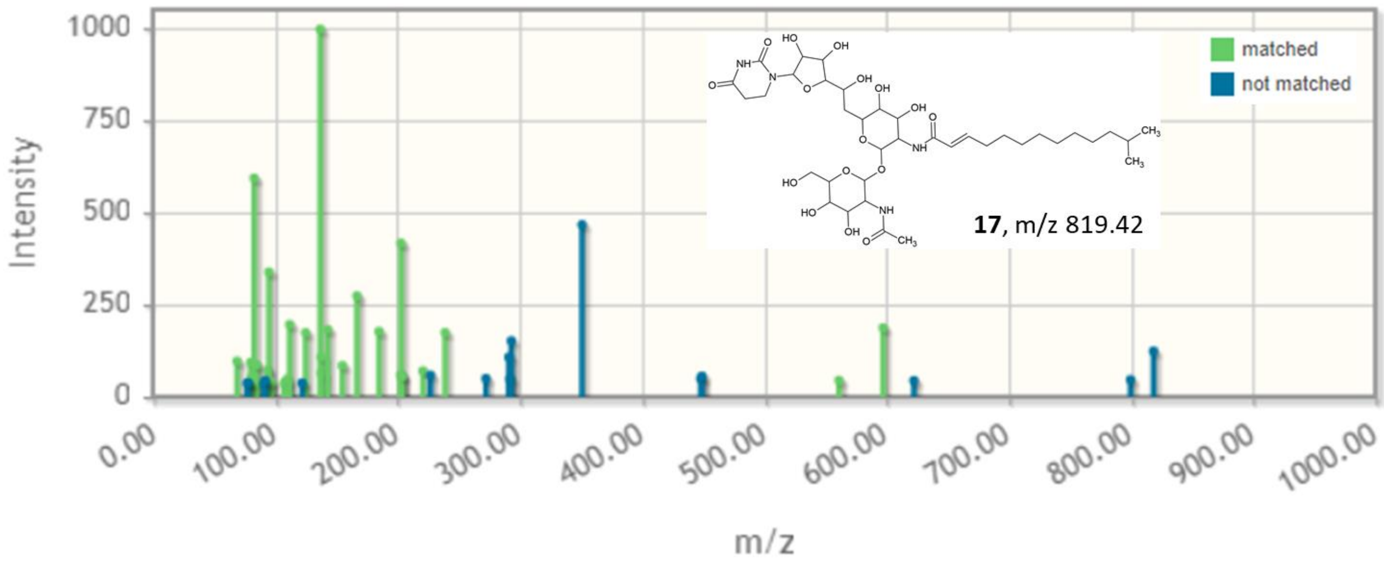

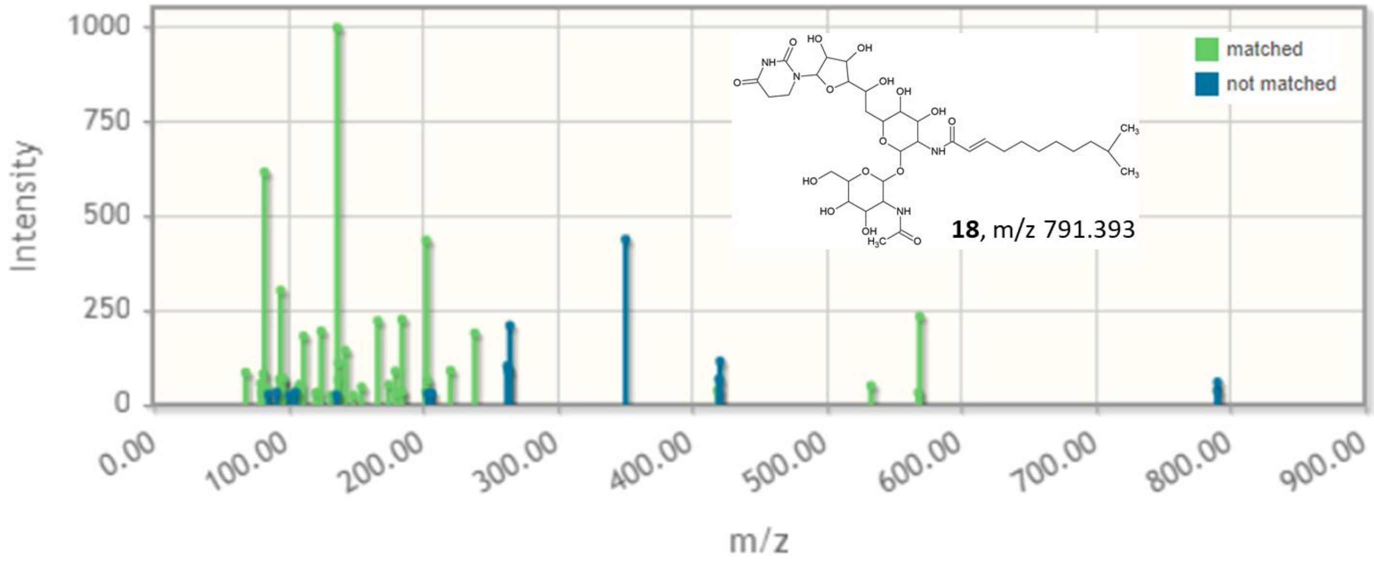

Figure S5: Comparison plots showing mass spectrometry fragmentation patterns of putative metabolites (19-21) from the polycyclic tetramate macrolactam molecular network. Peaks shared between predicted and experimental spectra are indicated in green and those not matched are in blue.

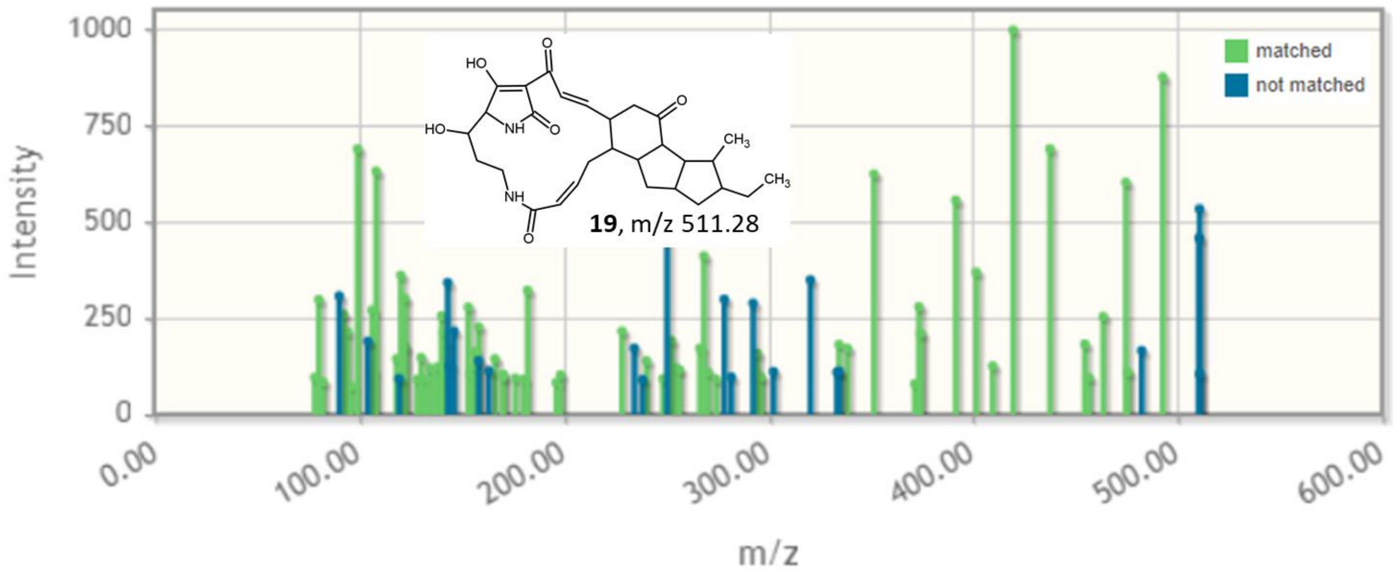

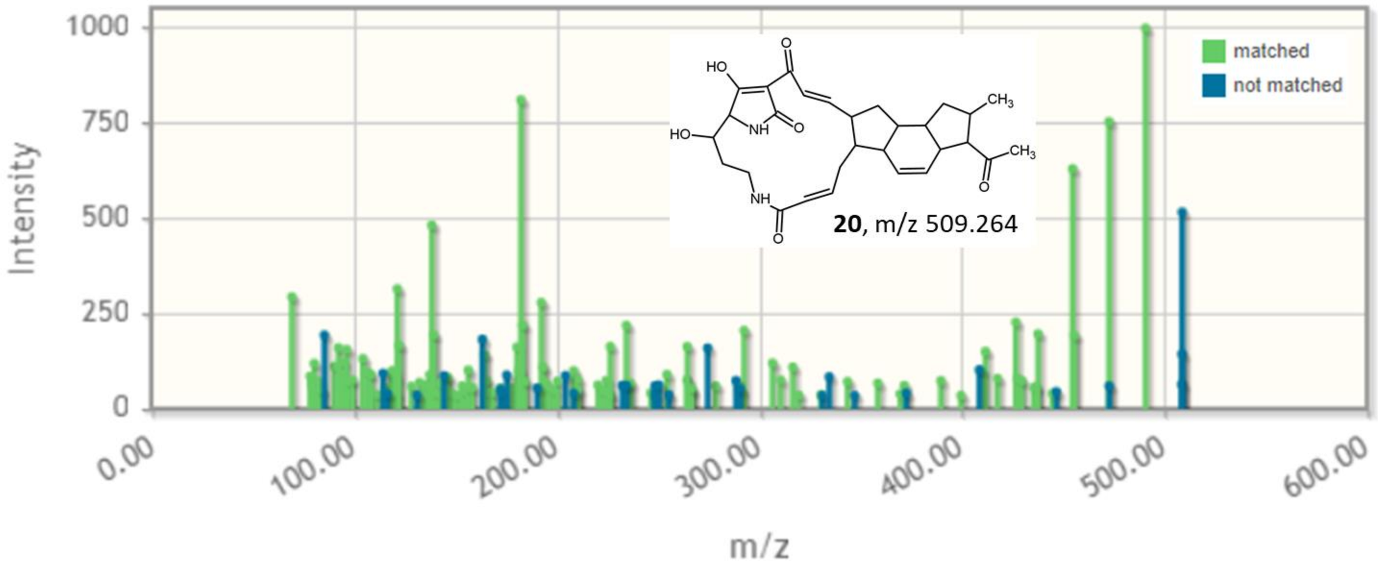

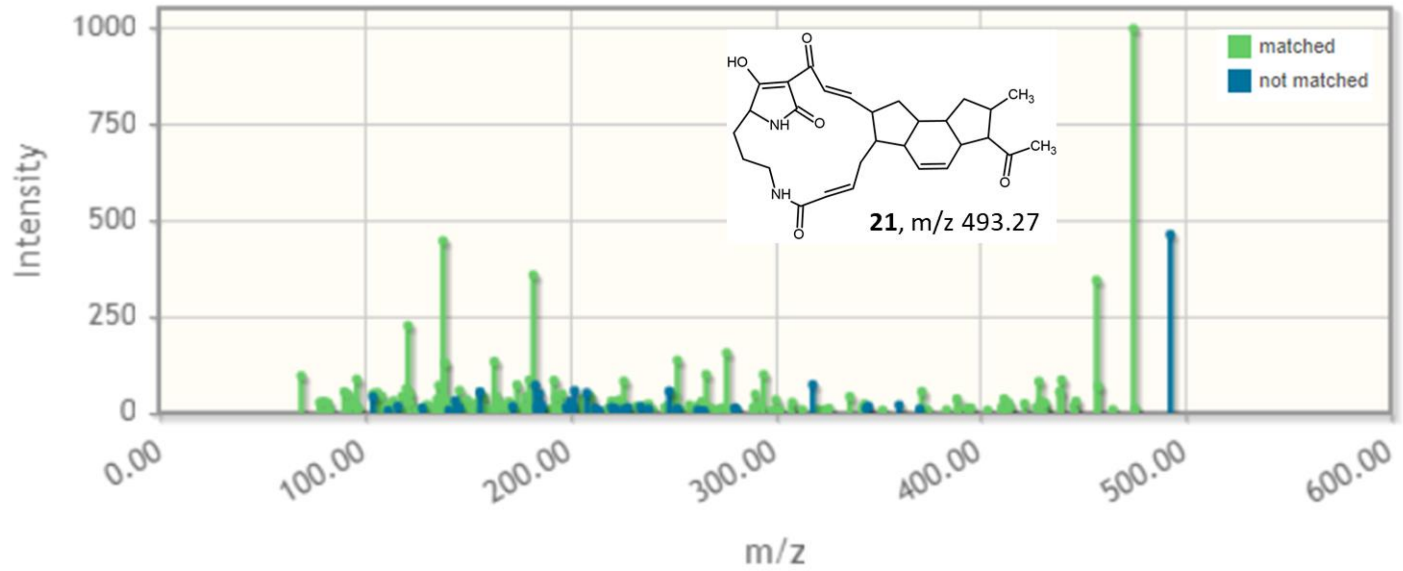

Intensity
1000
750
500
250
0
0.00
100.00
200.00
300.00
400.00
500.00
600.00
m/z
matched
not matched
HO
O
NH
NH
CH3
CH3
21, m/z 493.27

Figure S6: Comparison plot showing the predicted and experimental mass spectrometry fragmentation pattern of bafilomycin J (22). Peaks shared between the predicted and experimental spectra are indicated in green and those not matched are in blue.

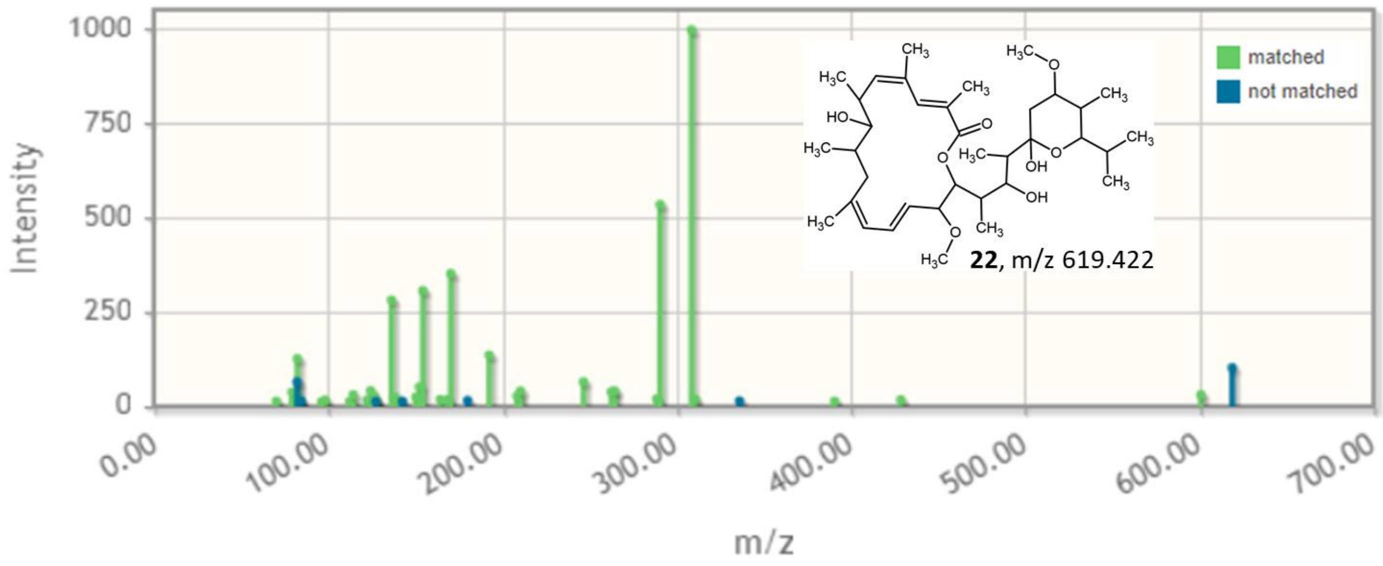

Figure S7: Comparison plots showing mass spectrometry fragmentation patterns of putative diterpenoid metabolites (23-25) from the current study. Peaks shared between predicted and experimental spectra are indicated in green and those not matched are in blue.

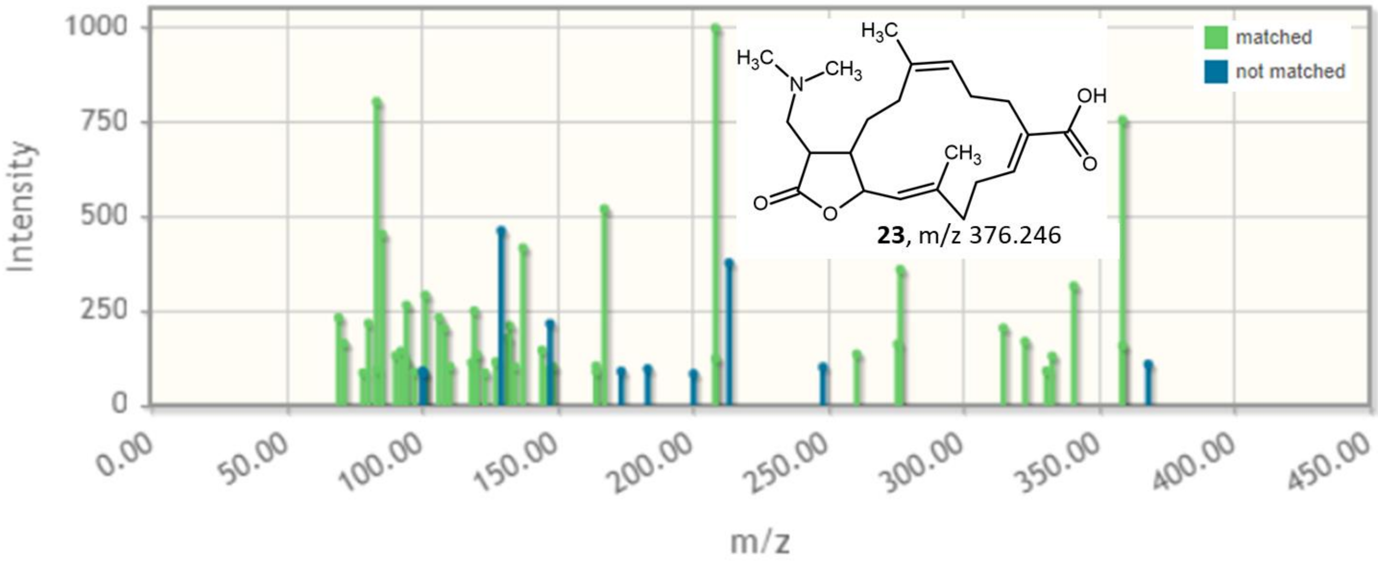

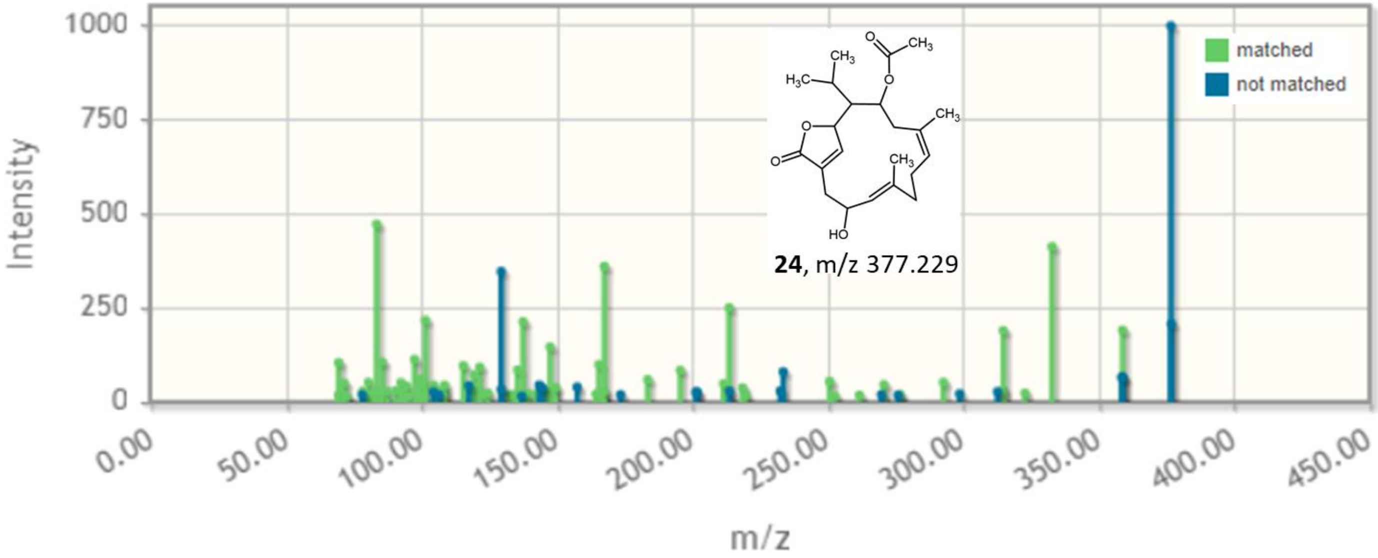

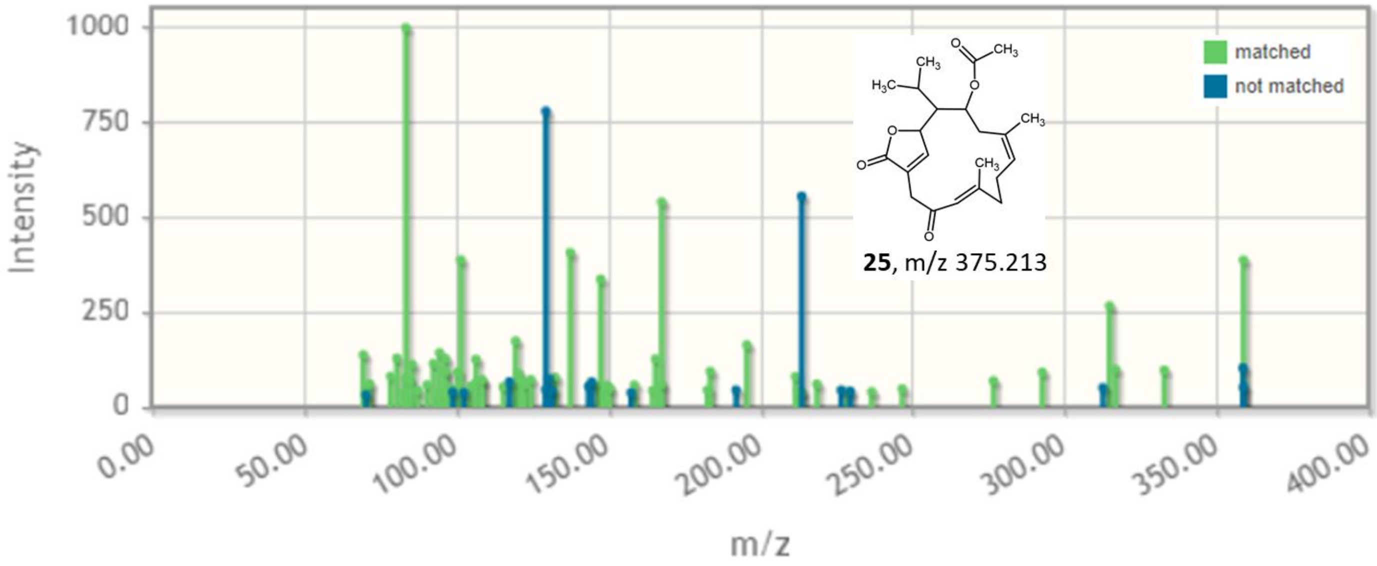
1000
750
500
250
0
Intensity
0.00
50.00
100.00
150.00
200.00
250.00
300.00
350.00
400.00
m/z
matched
not matched
25, m/z 375.213

Figure S8: Comparison plots showing mass spectrometry fragmentation patterns of putative organonitrogen metabolites (26-28) from the current study. Peaks shared between predicted and experimental spectra are indicated in green and those not matched are in blue.

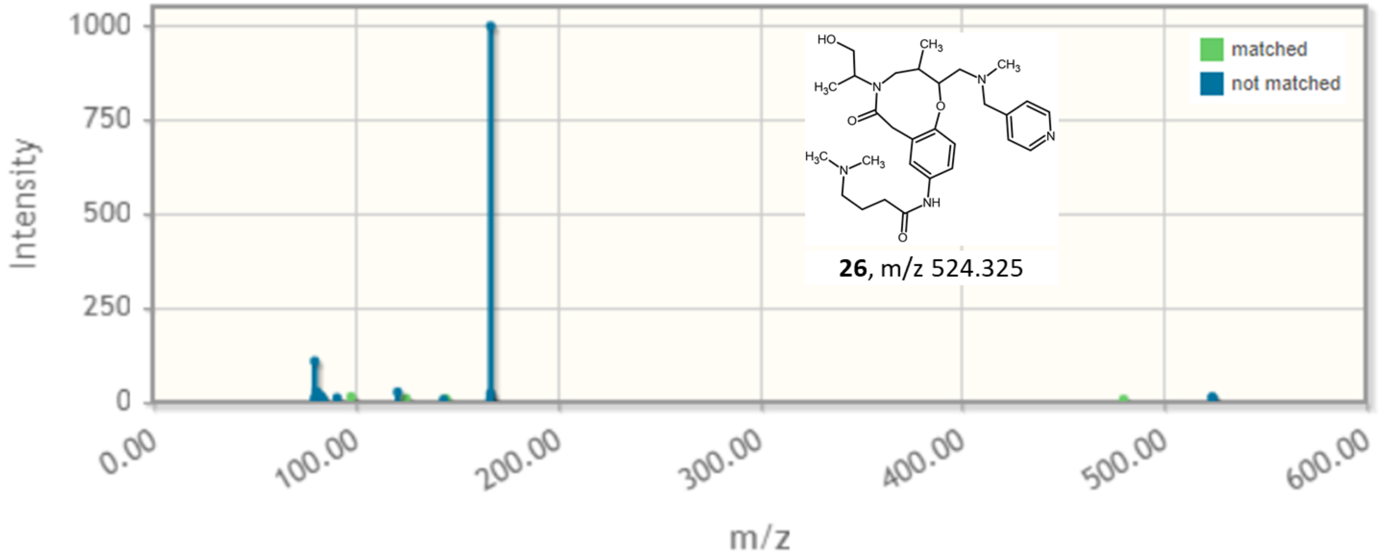

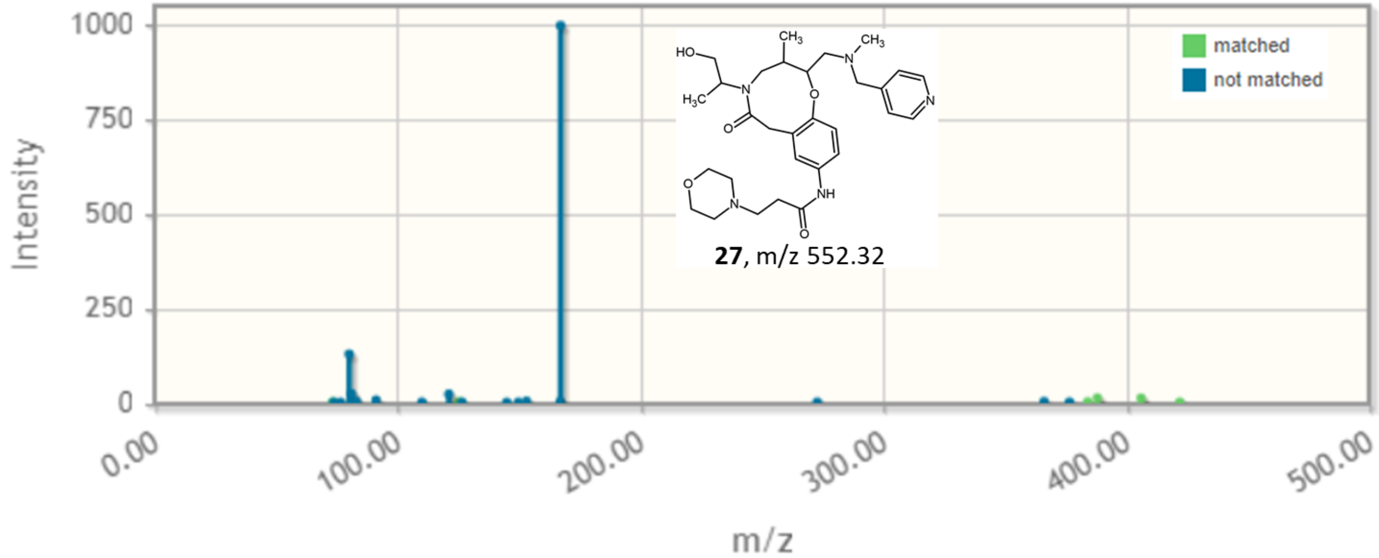

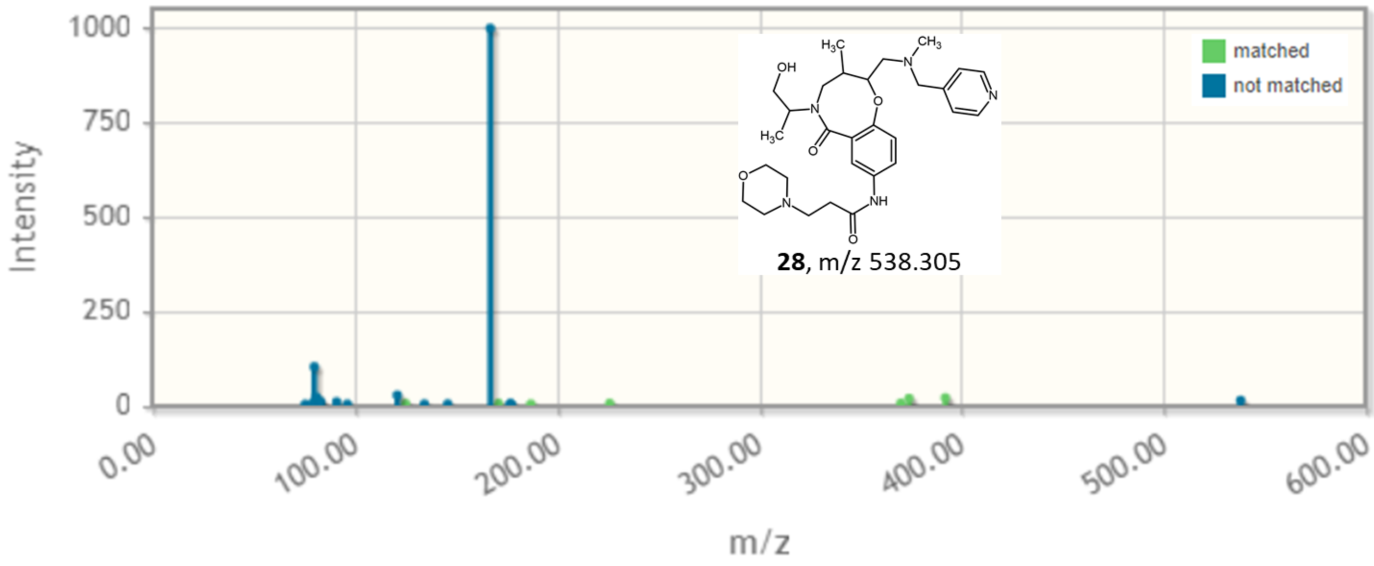

matched
not matched
Intensity
1000
750
500
250
0
0.00
100.00
200.00
300.00
400.00
500.00
600.00
m/z
28, m/z 538.305

Figure S9: The mevalonic acid-based plant triterpene biosynthetic pathway.

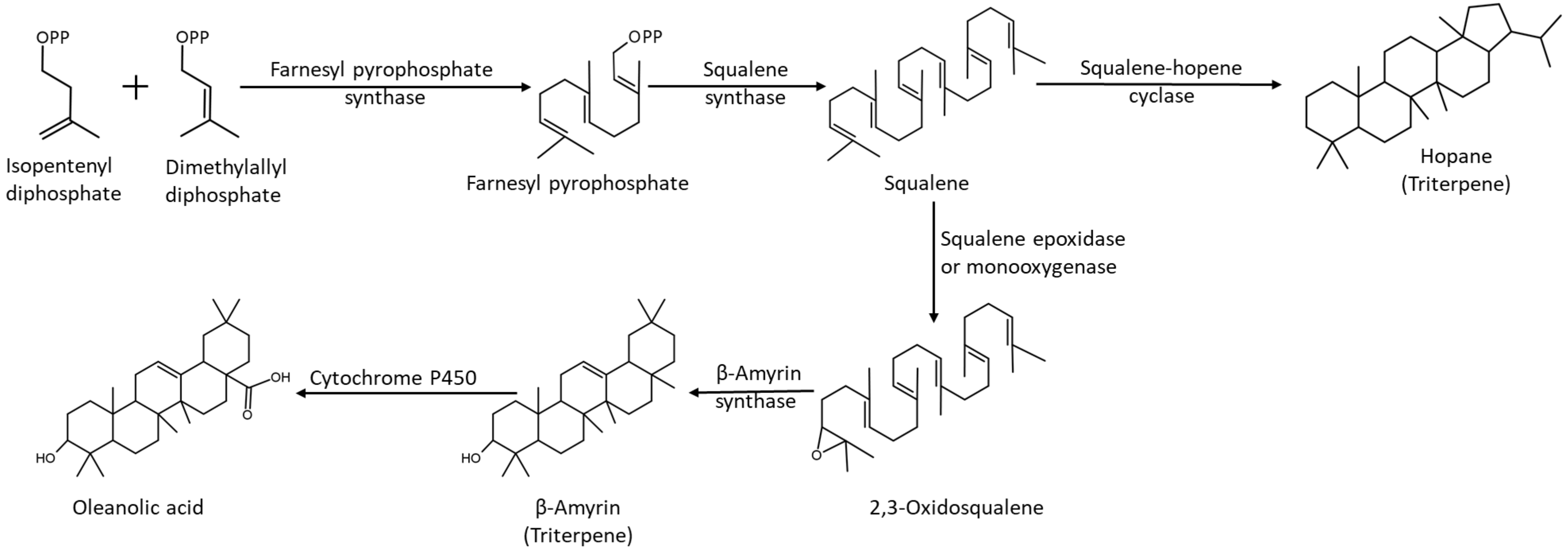

Figure S10: The proposed biosynthetic pathway for flavonoids and derived glycosides in *S. clavuligerus*. In the scheme leading to ferulic acid in *S. clavuligerus*, 4-coumarate-3-hydroxylase and caffeic acid *O*-methyltransferase are predicted to be encoded by *ncyP* and *SCLAV_5485*, respectively (shown in red), as their predicted gene products contain the required protein domains. In the current study, caffeic acid (shown in red) was also detected by GNPS in some of the *S. clavuligerus* overexpression strains (including *rpsL*-K88E).

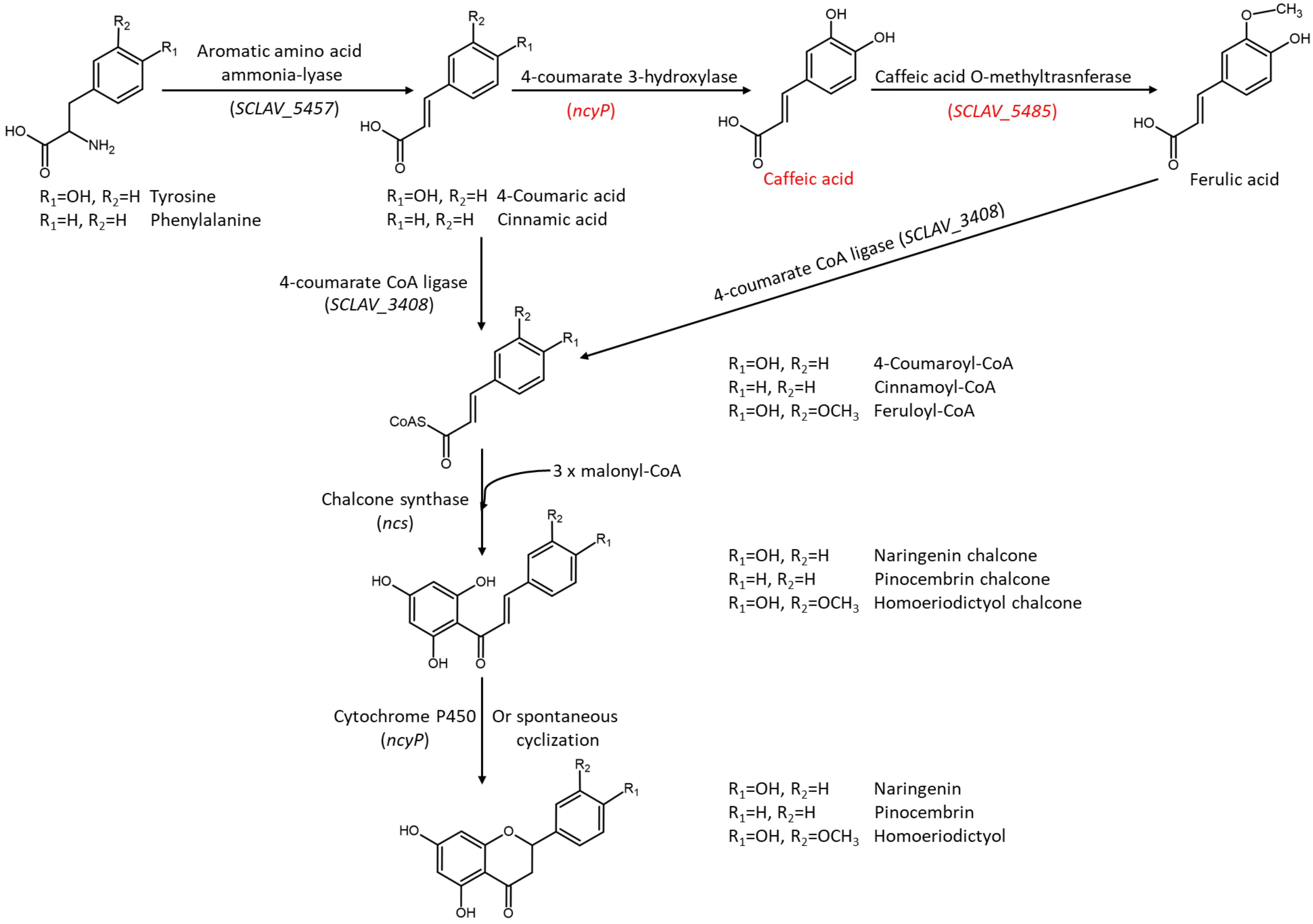

Figure S11: The proposed biosynthetic scheme for clifednamide A and B from ikarugamycin in *S. clavuligerus*.

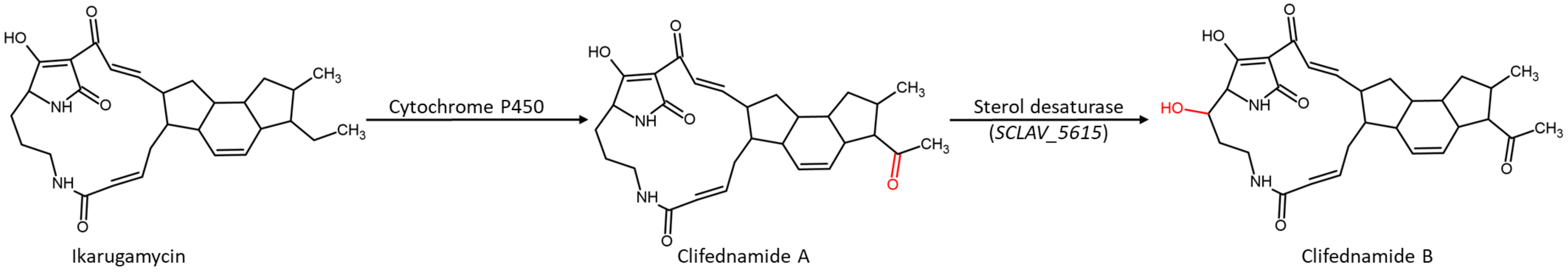

Figure S12: The proposed biosynthetic scheme for bafilomycin J involving putative products from a partial gene cluster present in *S. clavuligerus*.

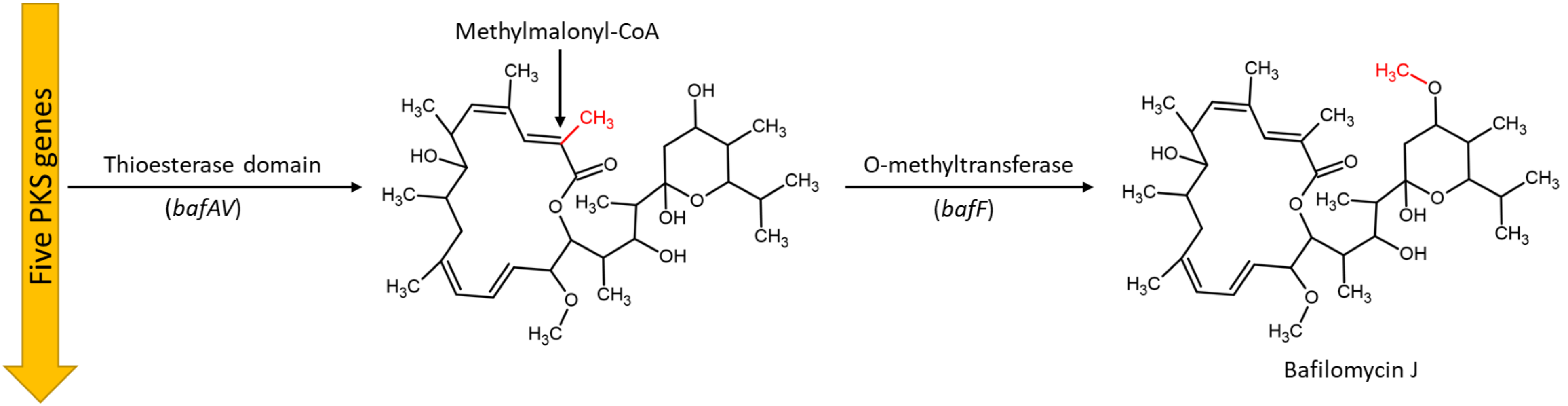

Table S1: Global natural products social molecular networking (GNPS) and network annotation propagation (NAP) parameters used for analysis in the current study.

GNPS parameters		NAP parameters	
Precursor ion mass tolerance	0.02 Da	Number of cluster index	0
Fragment ion mass tolerance	0.02 Da	Cosine value to sub-select	0.5
Minimum cosine value	0.6	Candidates for consensus score	10
Minimum matched fragment ions	3	Accuracy of exact mass candidate search	15 ppm
Network TopK	10	Acquisition mode	Positive or negative
Minimum cluster size	1	Adducts (positive)	[M+H], [M+Na], [M+NH_4], [M+K]
Run MSCluster	Yes	Adducts (negative)	[M-H]
Maximum connected component size	100	Multiple structural databases used*	GNPS, HMDB, SUPNAT, ChEBI, DRUGBANK, FooDB
Filter precursor window	Filter	Skip parent mass selection	No
Filter library	Filter	Maximum number of candidates structures in the graph	10
Filter peaks in 50Da window	Don't filter	Workflow type	Standard

* GNPS: Global Natural Products Social molecular networking (https://gnps.ucsd.edu/ProteoSAFe/static/gnps-splash.jsp)
HMDB: Human Metabolome Database (https://hmdb.ca/)
SUPNAT: SuperNatural II database (http://bioinf-applied.charite.de/supernatural_new/)
ChEBI: Chemical Entities of Biological Interest (https://www.ebi.ac.uk/chebi/)
DRUGBANK: https://go.drugbank.com/
FooDB: Food Database (https://www.foodb.ca/)
